# Physical Activity in Community Dwelling Older People: A Systematic Review of Reviews of Interventions and Context

**DOI:** 10.1371/journal.pone.0168614

**Published:** 2016-12-20

**Authors:** Olawale Olanrewaju, Sarah Kelly, Andy Cowan, Carol Brayne, Louise Lafortune

**Affiliations:** Cambridge Institute of Public Health, Forvie site, Robinson Way, CB2 0SR, Cambridge, United Kingdom; Universidade Federal do Rio de Janeiro, BRAZIL

## Abstract

**Background:**

The promotion and maintenance of higher physical activity (PA) levels in the older population is an imperative for cognitive and healthy ageing but it is unclear what approaches are best suited to achieve this for the increasing number of older people living in the community. Effective policies should be informed by robust, multi-disciplinary and multi-dimensional evidence, which not only seeks what works, but in ‘what context? In addition to evidence on the efficacy and effectiveness of PA for maintaining cognitive health, social contexts such as ‘how do we actually get older people to partake in PA?’ and ‘how do we sustain that activity long-term?’ also need highlighting. This review is part of a comprehensive evidence synthesis of preventive interventions in older age, with a focus on healthy behaviours to identify evidence gaps and inform policy relating to ageing well and cognitive health. An overview of systematic reviews of PA was conducted to explore three topics: (1) PA efficacy or effectiveness for primary prevention of cognitive decline in 55+; (2) Interventions efficacious or effective for increasing PA uptake and maintenance in 55+; (3) barriers and facilitators to PA in 55+.

**Methods:**

Multiple databases were searched for studies in English from OECD countries between 2000 and 2016. Quality of included reviews in questions (1) and (2) were assessed using AMSTAR. Review protocols were registered on PROSPERO (CRD42014015554, 42014015584, CRD42014015557) and reviews follow PRISMA guideline.

**Findings:**

Overall, 40 systematic reviews were included. Question 1 (n = 14). 8,360 participants. Evidence suggests that PA confer mild positive effects on cognition in older adults with and without previous cognitive impairment. However, there is insufficient evidence of a dose-response relationship. Evidence on the effects of PA on delay of dementia onset is inconclusive. Question 2 (n = 17). 79,650 participants. Evidence supports the effectiveness of a variety of interventions, including group delivered, centre-based and cognitive approaches on short-term uptake of PA behaviour. Question 3 (n = 9). 22,413 participants. Barriers include health status, previous PA habits and experiences, and cultural sensitivity, while facilitators include enjoyable activities and convenient scheduling.

**Conclusion:**

PA can offer small benefits to brain health, but evidence on how much activity is required to produce this effect is lacking. Evidence on the effectiveness of PA for preventing dementia and cognitive decline is lacking. Behavioural (walking, exercise) and cognitive (counselling and motivational interviews) interventions are effective for short-term uptake of physical activity in older people. In order to maintain long-term participation in PA, individualised interventions modelled using behavioural theories may be required. Public health messages should be aimed at promoting acceptable levels of PA above normal daily activities in older people. Policy and strategies aimed at increasing PA in older people should be encouraged while considering barriers and facilitators to behaviour change.

## Introduction

Populations globally are ageing and life expectancy in high-income countries is increasing [1]. In the United Kingdom, there are about 10 million people aged over 65, a number expected to rise by 1·1 million by 2020 [2]. But more older adults are living with disabilities and preventable health conditions now than ever before [3]. In response, there has been a growing emphasis in health policy on risk reduction approach to preventable diseases through life-style modification [4]. Research evidence on the importance of physical activity (PA) for the prevention and delay of long-term conditions such as osteoporosis, type 2 diabetes, obesity, stroke and coronary heart disease is strong [5–7]. Physical inactivity is estimated to contribute the highest population-attributable risk factor for Alzheimer’s disease (AD) and is associated with 20% of AD cases in the UK, USA and Europe [8]. There is also evidence of mild, short-term positive effect of PA on brain health [9, 10]. However, the effectiveness of physical activity interventions for the primary prevention of dementia and delay in the onset of cognitive decline is unclear.

Despite the well-known health, social and economic benefits of physical activity, inactivity remains a global challenge and policy response has not gained traction as expected [11]. In addition, physical activity rates decline substantially over the life course, particularly in late-life [12]. The promotion and maintenance of higher PA levels in the older population is an imperative for cognitive and healthy ageing but it is unclear what approaches are best suited to achieve this for the increasing number of older people living in the community. Effective policies should be informed by robust, multi-disciplinary and multi-dimensional evidence, which not only seeks what works, but in ‘what social context [13]? In addition to evidence on the effectiveness of PA for maintaining cognitive health, social contexts such as ‘how do we actually get older people to partake in PA?’ and ‘how do we sustain that activity long-term?’ also need highlighting. This review is part of a comprehensive evidence synthesis of preventive interventions for optimal cognitive health delivered in older age groups, with a focus on healthy behaviours to identify evidence gaps and inform policies relating to ageing well and cognitive health. We conducted a systematic review of reviews to explore three complementary questions:

1. What is the efficacy or effectiveness of physical activity for the primary prevention or delay of dementia or cognitive decline in older age (+55 years)?
2. What individually targeted interventions are effective or efficacious for increasing the uptake and maintenance of physical activity in older age (+55 years)?
3. What are the barriers and facilitators to the uptake and maintenance of physical activity in older age (+55 years)?

## Methods

The review protocols were registered on PROSPERO (CRD42014015554, 42014015584, CRD42014015557) [14–16] and reviews follow PRISMA guideline [17].

### Types of Studies

For questions (1) and (2), we included systematic reviews of any type of intervention studies. For question (3), systematic reviews that reported qualitative data specific to barriers and facilitators to PA were included. Only reviews published from year 2000 onwards in English from countries of the Organisation for Economic Co-operation and Development (OECD) were included.

### Participants/ Population

Reviews that predominantly included populations with a mean age of 55+, living in the community and in healthy condition, with pre-conditions such as high blood pressure, high cholesterol, overweight or obesity or people on medication were included. Reviews from disadvantaged populations, minority groups and vulnerable communities were included. Reviews focused on populations with previous ill health such as stroke, coronary heart disease, asthma were excluded.

### Interventions / Exposure

We included systematic reviews, which focused on interventions targeting PA and / or issues that prevented or motivated PA. Interventions which included prescription drugs, medication, and use of dietary supplements; management of existing disability and non-communicable diseases management of obesity; national policies, laws and taxation; screening and vaccination were excluded.

### Comparators / Control

Studies with any comparator or no comparator.

### Primary outcomes

**Question 1:** Primary outcomes were: measures of effectiveness related to prevalence, incidence or level of dementia or cognitive decline as measured by any appropriate measure including cognitive tests, scans and imaging and biomarkers.

**Question 2:** Primary outcomes were measures of effectiveness in older age related to the uptake or maintenance or change in PA levels and / or delivery / design of interventions such as setting and mode of delivery.

**Question 3:** We included primary qualitative outcomes from studies conducted in people aged 55+ related to issues for people in older age that prevent or limit or which help and motivate them to take up and maintain PA that may impact on healthy ageing, including settings, mode of delivery and personal issues.

### Searches

This review formed part of a comprehensive review, which used a wide range of search terms covering the following concepts and domains: ageing and older people; healthy behaviours and risk reduction relating to diet, physical activity, inactivity, alcohol, smoking; risk reduction relating to loneliness and isolation (i.e. leisure activities, participation), sun exposure, hearing and vision. Databases searched between 2000 and 2016 include: MEDLINE, EMBASE, PsycINFO, CINAHL, Social Science Index, the Cochrane Collaboration and Database of Systematic Reviews, Database of Abstracts of Reviews of Effects (DARE), HTA, reference list of included reviews and York CRD databases.

### Data extraction, selection and coding

Titles and abstracts were screened independently by two reviewers (from OO, SK, SM). Differences between reviewers’ results were resolved by discussion and when necessary in consultation with a third reviewer (LL). If after discussion, there was still doubt about the relevance of a study for the review it was retained. Full paper copies were obtained for all reviews identified by the title/abstract screening. Full paper screening was conducted independently by two people (from OO, SK, SM). We extracted data on study design; population; intervention details, setting and delivery; comparators; type of outcome measures reported; outcome measures (measures of uptake and maintenance of healthy behaviour); design/delivery of interventions and quantitative or qualitative data relating to implementation issues, barriers or facilitators) and results.

### Quality Assessment

The methodological quality of systematic reviews in Reviews (1) and (2) were assessed using the AMSTAR tool (www.amstar.ca). A minimum of 10% of the reviews was fully and double quality assessed. Any discrepancy between reviewers was resolved by discussion. Reviews that adequately reported at least eight of the possible eleven AMSTAR criteria were rated as high quality; between five and seven criteria as moderate quality; reviews reporting fewer than five criteria adequately were considered to be of low quality.

### Data Synthesis

Findings were narratively synthesised and presented. Findings were initially tabulated to map the evidence in terms of included studies, country, age, population, interventions, comparators, outcome measures and effectiveness to map the level of evidence, quality and gaps. Barriers and facilitators to PA are presented and discussed in later sections using the Andersen behavioural model of health service utilisation [18].

### Subgroup analysis

Data specific to frail older population and at risk of chronic health conditions were assessed and summarised separately where sufficient data was available.

## Results

**Fig 1:**
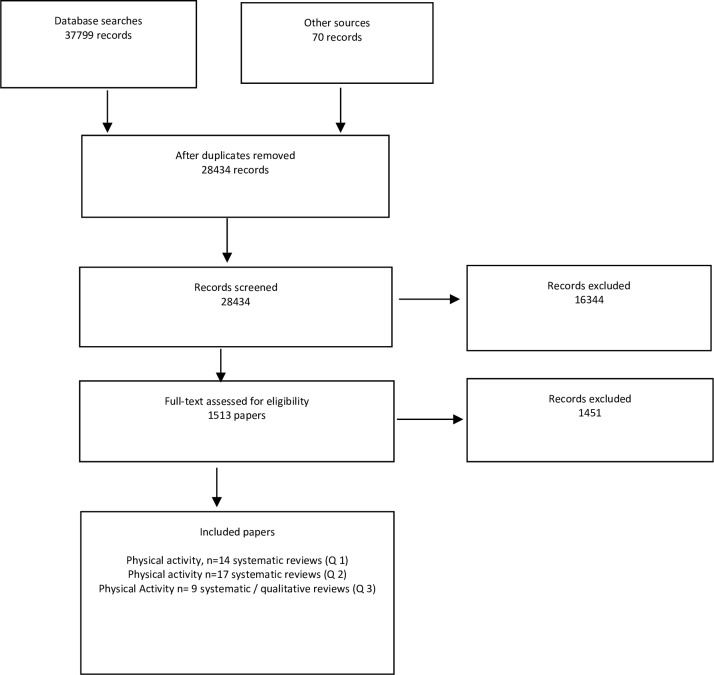
PRISMA flow diagrams for three review topics of all behavioural interventions including physical activity. Records included systematic reviews of intervention and qualitative studies.

The overall searches yielded 28,434 records, after 9,365 duplicates were removed. Forty systematic reviews on physical activity behaviours met the inclusion criteria. The study identification flowchart is shown in Fig 1. A summary of the included reviews and some of their descriptive characteristics are presented in Tables 1–3. We have reported all instances where the reviews reported pooled effect sizes. Full data is presented in supplement.

**Table 1:** Included reviews for effectiveness of PA for delay of dementia onset and cognitive decline in older people

Reviews	Included Studies	Age (Years)	Population	Relevant Outcomes / Measures
Angevaren 2008	11 RCTs	Range = 55–91	Sedentary, frail participants with age-related illness	Cognitive function tests
Balsamo 2013	8 RCTs	Mean = 74·8	Mixed population of normal cognitive older adults and MCI, 1 only female study.	Cognitive function tests
Cai 2015	13 intervention studies (9 RCTs and 4 non-RCTs)	Range = 70–78	Community dwelling older adults with mild cognitive impairment	Cognitive function tests
Carvalho 2014	10/27 RCTs	Age ≥ 60	Mixed population (sedentary, independently ambulatory, living independently)	Cognitive function tests and MRI
Chang 2012	10 RCTs	Mean ≥ 65	Healthy adults without cognitive impairment or specific disease	Cognitive function tests
Coelho 2013	5 RCTS and 1 non randomised CT	Mean = 66.2	Older women (no-frail and pre-frail), MCI, glucose tolerance criteria for pre diabetes or newly diagnosed; patients with major depression and healthy	Peripheral serum and plasma BDNF (brain-derived neurotrophic factor) concentrations; cognitive function tests; depression scales
Colcombe 2003	18 intervention studies (4 non-randomised CTs, 1 pre post and 13 RCTs)	Mean ≥ 55	Community-dwelling, "normal" older adult; Sedentary	Cognitive function tests
Gates 2013	14 RCTs	Range = 65–95	Predominantly female; with cognitive impairment; frail elderly	Cognitive function tests
Kelly 2014	25 RCTs	Range = 65–84	Community dwelling older adults	Cognitive function tests
Ohman 2014	8/22 relevant RCTs	Range = 50–86	60% female; mean mini-mental status examination score of 24	Cognitive function tests
Patterson 2010	12 intervention studies (6 non-randomised CTs and 6 RCTs)	NR	NR	Cognitive function tests
Sherder 2014	8 RCTs (5 RCTs involving normal cognition, & 3 with cognitive impairment	Range = 55–73 for no cognitive impairment; 75–86 for participants with cognitive impairment.	NR	Cognitive function tests
Tseng 2011	12 RCTs	Mean = 71·5	Older adult participants with and without cognitive impairment	Cognitive function tests
Uffellen 2008	21 RCTs	Mean > = 55 years	Older adult participants with and without cognitive impairment	Cognitive function tests

PA = Physical activity; RCTs = Randomised Controlled Trials, NR = Not Reported, MCI = Mild cognitive impairment

**Table 2:** Included reviews for interventions effective for increasing uptake and maintenance of PA in older people

Reviews	Included Studies	Age (Years)	Population	Relevant Outcomes / Measures
Asikainen 2004	28 RCTs in total	50–65	Postmenopausal women	Mean drop out, mean attendance. Injury Rates
Clegg 2012	6 RCTs in total	Median age = 83 (range 78–88)	Frail older people; 987 participants	Completion and adherence rates, Timed Up and Go (TUG)
Chase 2013	20 RCTs	Range = 66·30–81·70	Community-dwelling older adults	Self-reported PA (e.g. Physical Activity Scale for the Elderly (PASE)), Pedometer and accelerometer
Chase 2014	53 two-grouped treatment versus control comparisons	Range = 68–88	Community-dwelling older adults	Effect sizes for varying objective and subjective outcomes of PA uptake were calculated
Con 2003a	43 primary studies	Mean = 60–77·2	Community-dwelling older adults	Overall PA and episodic exercise behaviour.
Con 2003b	17 RCTs	Mean = 65+	Community-dwelling older adults	Overall PA and episodic exercise behaviour (< = 6 months post-test). Exercise maintenance (> 6 months post-test)
Cyarto 2004	8 intervention (5 RCTs, 2 quasi-experiments, 1 pre-post	Range = 40–>90	Community-dwelling older adults	Questionnaires measuring PA, exercise logs, Accelerometers
de Vries 2012	3/18 RCTs relevant to PA uptake & maintenance	Range = 60–85	Community-dwelling older adults with impaired mobility, physical disability and/or multi-morbidity.	Self-reported PA, TUG, 6 minute walk test, 400 metre walk test.
Fairhall 2011	15 RCTs	60+	Community-dwelling older adults	Self-reported PA
French 2014	16/25 relevant Intervention studies (1 feasibility, 1 pre-post, 1 cluster RCT, 13 RCTs)	Mean = 69 (range = 60 to 84)	Community-dwelling older adults	Change in Physical activity measured in 'd' Cohen ES
Geraedts 2013	9/24 relevant studies (8 RCTs & 1 pre-post)	55+	Community-dwelling older adults	7- Day PA recall, accelerometer, adherence rate, compliance rate.
Hobbs 2013	21 intervention studies (3 cluster RCTs, 2 pre-post and 16 RCTs)	Mean = 60·7 (SD = 4·4; Range = 55–67·6)	Older adults at risk of chronic conditions	Self-reported PA and accelerometer
Muller 2014	16 intervention-studies (2 cluster RCTs, 2 quasi-experiments, 1 non-randomised CT & 11 RCTs)	50+	Healthy, community dwelling older adults	Self-reported PA and accelerometer
Neidrick 2012	8/11 relevant RCTs	50+	NR	Self-reported PA and accelerometer
Nigg 2012	14/18 relevant RCTs	55+	NR	Self-reported PA and accelerometer
Stevens 2014	6 RCTs (5 RCTs & 1 Cluster RCT)	Range = 65 to 74	Community-dwelling older adults	Self-reported PA; Time to reach target of > = 90 mins / week of Moderate to Vigorous PA; duration of walking and vigorous exercise.
Van der Bijj 2002	38 interventions studies (8 RCTs and 30 non-randomised CTs)	Mean = 51–88	Community dwelling, healthy and inactive older adults	Participation rates.

PA = Physical activity; RCTs = Randomised Controlled Trials, CTs = Controlled Trials, NR = Not Reported

**Table 3:** Included Reviews for barriers and facilitators to PA uptake in older people

Reviews	Included Studies	Age	Population	PA type
Barnett 2012	5 qualitative studies	NR	Retired (6 months–5·6 years)	Recreational PA
Boehman 2013	5 qualitative studies	50+	Community dwelling older people living independently in their home	Population-based falls prevention exercise programs
Bunn 2008	24 studies (4 RCTs, 1 survey, 1 cross-sectional & 18 qualitative)	55+	Older population	Falls prevention programme
Child 2012	12 qualitative studies	Older adults	Community-dwelling older adults	Falls prevention programme
Cunningham 2004	1 qualitative, 2 cross-sectional and 3 surveys	Seniors	Community dwelling seniors	PA
Devereux-Fitzgerald 2016	14 qualitative studies	65+	Independent community dwelling older people	PA
Dunsky 2012	6/7 relevant surveys	45+	Adults and older adults	PA and sports
Franco 2015	132 studies (missed qualitative, RCTs, cross-sectional)	60–89	Community dwelling (85%); long-term care facilities, assisted-living facilities and hospitals.	Structured exercise programmes, other forms of physical activity or combination of both.
Horne 2012	10 qualitative studies	60+	Older adults from South Asian communities	PA

PA = Physical activity; RCTs = Randomised Controlled Trials, NR = Not Reported

### Included reviews

Overall, forty systematic reviews published between 2000 and 2016 were identified and included. We checked for overlap in primary studies between systematic reviews to avoid double counting. Almost half of the included reviews did not report the countries in which their included studies were conducted. For the rest that did provide this data, all were from OECD countries, with the majority conducted in the USA and Western Europe. For question (1) effectiveness of PA for delaying cognitive decline, fourteen systematic reviews met inclusion criteria with a median of 11 (range = 5–25) included studies and 8,360 participants [9, 10, 19–30]. Ten systematic reviews included only randomised controlled trials (RCTs), while four reviewed RCTs and other intervention studies [21, 23, 27, 29]. Eight [9, 10, 22, 24–26, 28, 30], four [21, 23, 27, 29] and two [19, 20] reviews were of high, good and low quality respectively. For question (2) on PA uptake and maintenance, seventeen systematic reviews of randomised controlled trials (RCTs) met the inclusion criteria with a median of 13 (range = 3–53) RCTs and 79,650 participants [31–47]. Nine [31, 32, 37, 38, 40–42, 45, 47], seven [33–35, 39, 43, 44, 46] and one review [36] were of high, good and low quality respectively. For question (3) on barriers and facilitators to PA, nine qualitative systematic reviews met the inclusion criteria with a median of 10 (range = 4–132) primary studies and 22,413 participants [48–57]. Of these, five reviewed purely qualitative studies [48, 49, 51, 54, 56], while the rest included a mixture of studies [50, 52, 53, 57].

### Interventions

Thirty-one systematic reviews evaluated interventions either aimed at uptake and maintenance of PA or delaying cognitive decline. Types of interventions reviewed varied considerably between single and multi-component interventions. Information on dosage (i.e. frequency, intensity, time) was poorly reported or omitted (50% of reviews). Duration of interventions varied between 6 weeks and 90 months, while the length of follow-up post-intervention ranged from 2 weeks to 48 months.

### Population

Population sample were recruited from community settings including, but not limited to home, work place, community and day centres, sheltered housing and primary care. Of the forty included reviews, one was targeted to older post-menopausal women [31]. Three reviews specifically looked at frail older populations [27, 28, 32], and one focused on a minority ethnic South Asian population [54]. Eleven reviews included older populations with pre-conditions for later ill health such as high blood pressure and high cholesterol [28, 31], impaired cognitive function [9, 19, 25, 26, 29], mood disorder [21], impaired mobility and disability [37] and impaired glucose tolerance [27, 41].

### Question 1: The effectiveness of physical activity for the primary prevention or delay of dementia or cognitive decline in the older age (+55 years)

Reviews reported quantitative outcomes related to short term improvement (4 weeks to 52 weeks) in cognitive function, measured by cognitive testing [9, 10, 19–28], scans and neuro-imaging [10, 27]. None of the reviews reported primary studies, which measured incidence or prevalence of dementia as main outcomes.

### Physical Exercise

By physical exercise, we refer to planned, structured, and repetitive physical activity which has as a final or intermediate objective, the improvement or maintenance of physical fitness. Exercise could be supervised, unsupervised, and performed in a group or individually [58]. Ten of Fourteen reviews [9, 19–22, 25–27, 29, 30] evaluated the effectiveness of exercise on cognitive outcomes. Six reviews reported on a variety of exercise training (strengthening, aerobic, Tai Chi, flexibility, balance), one reviewed only resistance exercise training [20] and another reviewed only aerobic fitness training [21]. Overall, five high [9, 22, 25, 26, 30], three good [21, 27, 29] and one low quality [20] reviews reported minimal but varied positive effects on cognition in the older population. Another low quality review [19] reported evidence of ineffectiveness. The heterogeneity in type, composition and dose of exercise interventions, mode of delivery and cognitive outcomes measures used contributed to a wide variation in the effects reported.

Two meta-analyses reported that aerobic fitness training (Hedge’s g = 0·41, SE = 0·037, n = 52, p<·05) improved cognitive performance in healthy older adults and markedly in the executive processing region of the brain [21]. However, effect sizes ranged from 0·9 to +6·4 confirming heterogeneity. A good quality review reported that aerobic exercise increased Brain Derived Neutrophic factor (BDNF), a biomarker for functional brain recovery in older adults [27]. However, a few primary RCTs (n = 6) were reviewed making it difficult to draw a definite conclusion on effect.

#### Walking

One high quality systematic review [24] and meta-analysis of randomised controlled trials found that walking improved executive functions in cognitively intact sedentary older persons who had lived a sedentary life (d = 0·36, SE = 0·1, 95% CI = 0·16, 0·55, z = 3·56, P<0.001). No significant effect was found on executive functions in older adults with cognitive impairment (d = 0·14, 95% CI = -·36, 64, z = 0·35, P = 0·56). The number of primary studies reviewed were limited (n = 8).

#### Dose-Response

No systematic review has reported evidence on the dose-response or threshold effect of exercise on cognitive function in the older population. Inconsistencies and variation in reporting the dose and parameters of exercise used in primary RCTs may have contributed to the difficulty in ascertaining these effects.

#### Mild Cognitive Impairment (MCI)

Three high and one good quality systematic reviews reported evidence of minimal effectiveness of exercise on cognitive functions in the older population with mild cognitive function [9, 22, 26, 29]. Ohman [22] and Uffellen [26] reported positive effects on one or several domains of cognition including global cognition, executive function and attention. Gates et al (2013) reported some evidence of positive effect on verbal fluency (ES = 0·17; 95% CI = 0·04, 0·30), global cognition [(ES = 0·74; 95% CI: 0·43, 1·05), (ES = 0·56; 95%CI: 0·19. 0·92), (ES = 0·69; 95%CI: 0·03, 1·32)], and memory [(ES = 3·37, 95%CI: 2·27,4·74), (ES = 0·54: 95%CI: 0·02, 1·08)]. Aerobic and combined exercises were reported to be the most effective for improving cognitive function in older adults with MCI. In contrast, Gates et al (2013) reported that aerobic exercise did not improve executive function. Overall, systematic reviews reported limitations in the primary studies reviewed such as small sample sizes, poor quality, and heterogeneity in interventions and outcome measures.

### Question 2: Effective Interventions to promote uptake and maintenance of PA in older age (+55 years)

#### Physical Activity (PA) Specific Interventions

We defined PA-specific interventions as those that involve carrying out only observable physical action, that is bodily movement produced by the contraction of skeletal muscle and that substantially increases energy expenditure. They include but are not limited to leisure-time PA (e.g. dancing, swimming), non-leisure (occupational and household), and exercise (e.g. aerobic, strengthening, flexibility). Five reviews [31, 32, 36, 37, 45] identified RCTs, which used PA as a stand-alone intervention to improve the uptake and maintenance of PA levels among older adults. There was mixed evidence from these reviews on the effectiveness of PA-specific interventions for improving PA uptake and maintenance. In addition, heterogeneity in reported outcome measures, population and settings made it difficult to extrapolate the effectiveness and / or overall effect size of PA from individual studies.

Four reviews reported positive effects and association with PA levels. One high quality review [31], which identified twenty-eight RCTs found high attendance (mean 84%), low dropout (mean 13%) and low injury rates (mean 3%) for walking intervention. However, these results were from interventions of short term duration (< = 1 year) in post-menopausal older women only. Another high quality review [32] found completion rates of home-based exercise for frail older people to be generally high (median 83%, range 65–88%). It concluded that interventions of shorter duration generally had higher completion rates compared with those of longer duration. In addition, this review reported high rates of adherence to exercise interventions. One high quality review [45] reported that tailored PA interventions significantly increased PA levels.

One review [36] reported positive effect of progressive resistance training among older adults. But, this review did not adequately report vital primary studies characteristics such as comparators, length of follow-up, intervention dose, and outcome measurement. Conversely, one high quality review [37] reported ineffective findings on physical exercise therapy aimed at improving PA levels in the frail elderly. This may be because only three out of eighteen RCTs reported on PA levels, of which two studies (SMD: 0·08, 95% CI: -0·21, 0·31, I^2^: 0%) were pooled in the meta-analysis.

#### Non-PA Interventions

These included studies of interventions to increase PA uptake, which do not involve any observable or participatory PA. Three good quality reviews [33, 43, 46] identified and reviewed non-PA interventions, which mostly included health education, counselling, goal setting, motivational interviewing and other behavioural change techniques. All reviews reported that their interventions were effective for improving uptake of PA in the short-term. However, none of these reviews reported a long term intervention effect on PA behaviours in older adults.

#### Multi-Component Interventions

Four [33, 38, 44, 47] reviews identified studies, which investigated multi-component interventions aimed at promoting the uptake and maintenance of PA in older adults. The components of reviewed multi-component interventions varied between intervention studies. Two reviews [33, 38] examined studies, which combined cognitive and behavioural interventions components including supervised exercise, telephone prompting, telephone counselling, face to face activity planning, goal setting and health education. A good quality review [44] included primary studies, which evaluated a combination of physical activity and nutrition related behaviours.

Two reviews [33, 44] reported inconclusive findings on the effect of multi-component interventions on short-term PA behaviour in older adults. Although the third review [38] and meta-analysis reported that multi-component behavioural intervention with an exercise component had a larger effect (Hedges’ g = 0·25, 95%CI = -0·04, 0·53, P = 0·09) on participation than exercise intervention alone (Hedges’ g = 0·09, 95%CI = -0·01, 0·19, P = 0·07), the difference was not statistically significant (Hedges’ g = 0·22, 95% CI = −0·05 to 0·50, P = 0·10). One review of combined behavioural and cognitive interventions reported varied but positive long-term effects (range between 9 to > 12 months) on PA behaviour in older adults [33].

#### Delivery (Mode and Settings)

Eight reviews [34–36, 41–43, 45, 46] identified and provided evidence on the association between settings, mode of intervention delivery and uptake of PA among older adults. Three reviews (one good, one high and one low quality) reported that interventions delivered in primary care centres and general practices can produce at least short term increases in physical activity [36, 43, 45]. However, there is limited evidence to evaluate whether long-term changes can be achieved. Tailored interventions may be effective for increasing uptake of PA among older adults. Two high quality reviews reported that using tailored interventions were more effective in increasing PA level when compared with generic interventions [41, 45].

Two good quality reviews [34, 46] reported positive findings on the effectiveness of interventions delivered in group format. One review of twenty-eight studies reported that group-based PA interventions increased short term participation by a mean of 84% (range = 55%-100%) [46]. This review also reported that participation in group based interventions declined after one year. The second review [34, 59] and meta-analysis reported that studies testing centre based exercise reported significantly larger effect sizes (d_wc_ = ·47± 16, k = 15) than studies with home-based exercise (d_wc_ = ·24± 08, k = 28). The effect sizes (d_wc_) were calculated using standardised mean differences in PA scores weighted by sample size. The review findings suggested that older adults who exercised at centres in comparison with home based activity were much more likely to continue PA. There was conflicting evidence on the effectiveness of mode of delivery of interventions such as face to face, contact with a professional and non-face to face. One high quality [42] review reported that non-face to face interventions were effective in the short and long-term for improving PA levels in older people (1 week to 24 months). However, there was one high quality meta-analysis [41], which suggested that the mode of delivery was not necessarily important for intervention effectiveness. It should be noted that the latter study [41] based its findings on two RCTs.

#### Intervention dose / intensity

Three reviews [41, 42, 59] reported mixed evidence on the effects of intervention dose / intensity on the uptake of PA among older adults. One good meta-analysis reported that intense direct contact with activity professionals doubled the effect size of the interventions [59]. Conversely, a high quality meta-analysis reported ineffective findings on the effect of intervention intensity [41]. Interventions that had more intervention contacts (≥ 11 contacts) did not have a detectable effect on PA levels at 12 months (SMD = 0·20, 95% CI = -0·08, 0·47, I^2^ = 71%), while interventions with fewer contacts (< 11 contacts) had a positive intervention effect (SMD = 0·16, 95% CI = 0·06, 0·27, I^2^ = 38%). A third high quality review reported a positive dose-response effect of non-face to face interventions aimed at improving PA uptake [42]. However, one out of its six primary studies reviewed reported a negative intervention effects for newsletters.

#### Interventions with theoretical underpin

Behavioural interventions such as physical activity are sometimes difficult to evaluate due to their complex nature. Some studies use theoretical models as a basis for developing effective behavioural interventions in order to understand their likely mechanism of effect. Three good [33, 34, 39] and three high quality reviews [41, 42, 47] discussed the use of behavioural theories in developing interventions and their associations with PA uptake / maintenance. More than 68% of the primary studies included in these six reviews reported the use of behavioural change models and related theoretical frameworks. Behavioural models used include Social Cognitive Theory [39, 42] and Trans-theoretical Model [41, 42], Health Belief Model [39], Kanfer’s Self-Control Model [39], Pender’s Health Promotion Model [39]. Four reviews [34, 39, 41, 42] including one meta-analysis reported positive associations between theory-based interventions and short term PA uptake (d = 0·14, 95% CI 0·09, 0·2, p<0·001). One good quality study reported that behavioural change techniques such as motivational interviewing and goal setting were useful for enhancing long-term (≥ 2 years follow-up) PA behaviour change [33].

#### Subgroup analysis

The uptake of physical activity in a subgroup in people considered frail or at risk of chronic conditions was discussed in three systematic reviews. Two high quality reviews [32, 37] reported on the effect of behavioural interventions on PA uptake among the frail elderly while one good quality review focused on older adults with preconditions for later ill health such as high blood pressure, impaired glucose tolerance and high cholesterol. The first high quality review [32] reported a high adherence and completion rate of home based exercises among the frail elderly. It also reported that interventions of shorter duration generally recorded higher completion rates compared to those of longer duration. The second high quality review [37] reported did not find the use of physical exercise effective in the frail old. A good quality review [41] reported that multi-modal interventions helped by behavioural cognitive techniques were useful for increasing short term PA uptake among those at risk of chronic conditions such as impaired glucose intolerance, hypertension and obesity. None of these studies reported intervention effects on long-term PA maintenance.

### Question 3: Barriers and facilitators to the uptake of physical activity in older age (+55 years)

Nine qualitative reviews explored barriers, facilitators and influences of physical activity (PA) uptake among older adults [48–54, 56, 57]. Three reviews [49–51] investigated barriers and facilitators (B&F) to the uptake of falls prevention exercise programmes, one review focused on recreational PA [48], one explored sports activities [53], while the final two explored PA in general [52, 54]. Two reviews explored the perceptions of older adults towards PA participation and the acceptability of PA interventions [56, 57]. One review explored facilitators and barriers among South Asian older adults [54], while another review explored PA uptake among older adults after retirement [48]. Although the systematic reviews often used different themes and categories to explain barriers and facilitators, the factors identified were similar across studies. A summary of barriers and facilitators found are presented in Table 4. The following sections will discuss barriers and facilitators frequently cited in our identified reviews using the Andersen behavioural model of health service utilisation [18, 60].

**Table 4:** Identified barriers and facilitators of PA uptake in older population categorised by predisposing, enabling and need factors

	Barriers	Facilitators
**Predisposing Factors**	Health status; previous PA habits; fatigue, low self-efficacy; low perceived value of recreational PA and preference for productive / meaningful PA; lack of motivation; body image, fear, lack of social support, family and household commitments; fatalism; stigma; collectivist attitudes; cultural sensitivity; language; previous exercise experience; cultural acceptability, underlying beliefs about personality type	New personal challenge, health; enjoying the activity; previous exercise experience; Social support, social contact, role models, Facilitative relatives; Group, peer and community support; Instructor support.
**Enabling Factors**	Environment (Light, crime, litter, noise, heavy traffic, footpaths safety, access to and convenience of facilities), time, poor access/awareness, cost/ finance,	Communication (positive reinforcement, information, language), time, customisation (tailoring of intervention, personalised modification), making exercise fun / enjoyable / sociable, good leadership/facilitation, motivation, Convenient scheduling/ reasonable pricing/good access and transport, facilitate feeling of ownership of interventions
**Need Factors**	NA	Referral from health-care professional (especially doctor)

#### Andersen Behavioural Model

This was created to empirically test hypothesis about inequality of access to health service in the United States of America. Andersen‘s model views access to service as a result of decisions made by an individual, constrained by their position in society and the availability of health services. The model postulates that predisposing, enabling and need factors predict the utilisation of health services. We use Andersen’s model to further understand the relationship between established barriers and the uptake of physical activity among the older population.

#### Predisposing Factors

Predisposing factors are based on an individual’s propensity to participate in PA. They include personal characteristics such as demographics, social structure, cultural norms, and beliefs. All except one review [53] identified predisposing factors of PA. The review of PA uptake among older adults after retirement identified health status, previous PA habits, and new personal challenges as predisposing factors for PA uptake. Reviews of PA with the objective of preventing falls [49–51] identified predisposing factors including health, motivation, feelings, fatalism, stigma and fear of falling. The predisposing factors identified in the review of PA uptake among South Asian community dwelling older adults included language, overprotective family, dependency on social support and group norms, cultural sensitivity and collectivist attitudes [54].

#### Enabling Factors

Enabling factors are based on the premise that even though an older adult is predisposed to participating (or not) in PA, certain factors must be in place to facilitate PA uptake. These generally include material resources (transportation, cost) and the availability of services (PA facilities / primary care centres). The review on South Asian older adults identified communication, information provision, source of advice, positive reinforcement as enabling factors to PA uptake among older adults [54]. Other enabling factors associated with the physical environment include safe footpaths, access and convenience of facilities, security, and lighting [52].

#### Need Factors

These intrinsically refer to those factors, which should necessitate the access or uptake of PA. This review’s remit is to explore barriers and facilitators in community dwelling older adults without previous ill health which would have excluded primary studies focused on health conditions requiring PA as intervention. Therefore it is not surprising that the reviews identified few need factors to promote the uptake of PA. However, two reviews [49, 50] identified referral or recommendation by a health professional / doctor as a facilitator of PA uptake. Referral by a health professional to a PA programme would normally indicate that there is a need for a patient to participate, either for preventative, or rehabilitative management.

## Discussion

This review found that several intervention modalities including PA-specific (walking, exercise), non-PA (education, counselling, and motivational interviewing), centre-based and group based interventions were effective for increasing short term uptake of PA among the older population. Frail older people adhered better to shorter duration exercise. The variety of effective interventions may have important implications for choice and availability of intervention in a heterogeneous population with different levels of ability and acceptability. Our findings suggest that a combination of behavioural and cognitive interventions underpinned by theory may be effective for long-term maintenance of PA. This is supported by a recent systematic review, which showed that individually tailored programmes underpinned by behavioural change techniques were effective at maintaining long-term PA levels in older adults [61]. However, reviewers consistently reported that there were an insufficient number of primary studies of long duration / follow up to show conclusive evidence on long term PA change [37, 38].

None of the identified reviews reported outcomes of reduced incidence / prevalence in dementia. This review found positive effects of physical exercise on cognition in the older population with normal cognition and mild cognitive impairment. However, there wasn’t conclusive evidence on the type of exercise that conferred the most cognitive benefit. No evidence was found relating to primary prevention of dementia or cognitive decline. We found evidence from one high quality review that walking was effective for improving executive function in sedentary older adults. This is particularly important because walking is a natural, routine aspect of most people’s daily activity and highly accessible. However, older adults who are already active may require higher intensity exercise to reap the cognitive benefit. There is no evidence on the dose-response or threshold effect of exercise on cognitive function in the older population, which is not helped by the under-reporting of information needed to determine such effect.

In addition, inconsistencies in outcome measures and intervention makes it difficult to pool results for meta-analyses, which is necessary to increase power and improve effect size [9, 10, 20, 23, 27]. This challenge could be partly addressed by agreeing standardised PA activities and core outcome measures for future dementia prevention trials. Other limitations reported by authors / reviewers included few RCTs and small sample sizes [19, 24], short duration [28], selection bias [10], external validity[10] and reporting of trials [20, 22]. A broad range of barriers and facilitators to the uptake and maintenance of PA among older adults were identified. Key barriers include health status, previous habits, low self-efficacy, time, access, and finance, perceived value of PA, environment. Key facilitators include enjoyment, personal challenge, social support, effective communication, information, access and convenient scheduling. Although key domains of importance are emerging from our reviews, including a previous one for mid-life [62] more needs to be learnt about context specific issues that would promote PA maintenance in older adults; including as they progress in age, key life transitions and cultural context.

One limitation of this review is that it focuses on studies from developed countries and may not be generalizable to developing settings. It is also an overview of reviews published in English language. The strength of this review is that rather than focusing on gaps in literature and research, we reviewed a range of issues, including effectiveness, mechanism of action, barriers and facilitators to inform the development and implementation of contextualised and tailored programmes for cognitive health and well-being in the older population [13]. Further, it is envisaged that this will underpin policies and commissioning of services relating to older people’s cognitive health and well-being.

## Conclusion and Recommendations

In an increasingly ageing population, knowing what works, and in what context is crucial for developing healthy ageing policies. In order for our older population to achieve meaningful and beneficial effects of PA, evidence–informed policies should be aimed at encouraging uptake and maintenance of PA. There is evidence that aerobic, strengthening exercise and walking (in sedentary subgroup) may confer minimal benefits to cognition in older people with and without existing cognitive impairment. Many interventions aimed at increasing PA level among older adults appear to be effective in the short-term and should be encouraged while considering barriers and facilitators to behaviour change. Predisposing behaviours and attitude towards PA may be more entrenched in older age, making it more challenging to alter when compared with middle age [62]. Therefore an argument could be made for government and local policies to encourage change in PA behaviour in older people by targeting enabling factors including better communication and information on the benefits of PA, affordability, and environmental safety. In addition, affordability could be addressed by helping the older population to activate PA-promoting local community assets such as walk-trails, green space, group activities and social networks [63, 64]. In the absence of evidence on the minimum PA that is effective for maintaining brain health and increasing participation, public health messages should be aimed at promoting acceptable levels of PA above normal daily activities in older people. Future research and trials should aim to address gaps including PA thresholds; dose-response; long-term effect; standardized outcomes measures and exploring PA behaviours in minority groups.

## Supporting Information

S1 Table Characteristics of included systematic reviews in question 1 (PA effectiveness for primary prevention of cognitive decline in 55+) (PDF)

S2 Table Characteristics of included systematic reviews in question 2 (Interventions effective for increasing PA uptake and maintenance in 55+) (PDF)

S3 Table Characteristics of included systematic reviews in question 3 (Barriers and facilitators to PA in 55+) (PDF)

S4 Table Quality assessment of SR on PA effect on cognition in older adults (PDF)

S5 Table Quality assessment of SRs on interventions to promote uptake of PA in older adults (PDF)

S1 Appendix Search Strategy (PDF)

S1 Protocol PROSPERO registered protocol for systematic reviews of interventions in older age for the primary prevention, delay of dementia and cognitive decline (PDF)

S2 Protocol PROSPERO registered protocol for systematic reviews of interventions in older age to increase uptake and maintenance of healthy behaviours that may impact on successful ageing (PDF)

S3 Protocol PROSPERO registered protocol for systematic reviews of barriers and facilitators to uptake and maintenance of healthy behaviours in older people (PDF)

S1 Checklist PRISMA checklist (DOCX)
