# Supplementary material for: Physical Activity in Community Dwelling Older People: A Systematic Review of Reviews of Interventions and Context
Source: PLoS One. 2016 Dec 20;11(12):e0168614. doi: 10.1371/journal.pone.0168614 (PMC5173028; doi:10.1371/journal.pone.0168614)
Supplement: S1 Table — (PDF) [file pone.0168614.s001.pdf]

S1 Table: Characteristics of included systematic reviews in question 1 (PA effectiveness for primary prevention of cognitive decline in 55+)

Study	Included Studies, Eligibility Criteria and Design	Country	Age	Population	Interventions	Control	Relevant Outcomes / Measures	Findings
Angevaren 2008	Older people (≥ 55 years) without cognitive impairment. 11 randomised clinical trials; Any PA intervention aimed at improving cardiorespiratory fitness; comparison interventions with no treatment, strength or balance and a program of social activities or mental activities. Participants recovering from surgical treatment; with comorbidities that precluded participation.	International	Age range (55-91)	Sedentary, frail participants with age-related illness	Cycling, walking, jogging, strengthening exercise, resisted, weight training, aerobic.	No activities, stretching exercise	**Outcomes:** Cognitive speed, verbal memory function (immediate), visual memory function, working memory, memory function (delayed), executive functions, perception, face recognition, cognitive inhibition, auditory attention, motor function. **Outcome Measures:** Ross Information Processing Assessment, Wechsler Adult Intelligence Scale (WAIS), Randt memory test story recall.	Overall, the effects of aerobic exercise on cognitive function compared with any other intervention was significant for speed (SMD random effects 0.26, 95% CI 0.04, 0.48, P=0.02), and visual attention speed (SMD random effects 0.26, 95% CI 0.02, 0.49, P=0.03). The effects of aerobic versus no intervention were positive for auditory attention (WMD random effects 0.52, 95% CI 0.13, 0.91, P<0.01) and motor function (WMD random effects 1.17, 95% CI 0.19, 2.15, P=0.02). Aerobic training appear effective in the short term for cognitive delay or prevention. Aerobic training was not superior to strength training.
Balsamo 2013	Older people with CI and AD. 8 RCT studies	USA, Australia, Brazil, France	Average age = 74.8 years	Mixed population of normal cognitive older adults, AD and MCI, 1 only female study.	Structured physical exercise, stretching, strength training, walking, daily living activities, strength training, cardiorespiratory training, music therapy	ADLs (bingo, patchwork, sewing), balance, stretching,	Outcomes: (Cognitive function) Executive, short-term memory, attention, long term episodic memory. **Outcome Measures:** ADAS-Cog, MMSE, Wechsler Adult Intelligence Scale III, Wechsler Memory Scale-revised, Toulouse-Pieron's concentration attention Test, Rey Osterrieth Complex Figure.	Although 5/8 studies showed higher cognitive response than controls, evidence was inconclusive due to lack of study power.

Cai 2015	13 studies (9 RCTs and 4 non-RCTs). Papers were included if: (i) participants were community-dwelling adults with the average age of 60 or older; (ii) participants had MCI; (iii) they reported experimental or quasi-experimental studies; (iv) physical exercise or physical therapy interventions were described; and (V) they directly measured cognitive performance as an outcome.	International (USA, Japan, Hong Kong, Australia, Canada, Brazil, Spain	Mean ages (70-78 years)	Community dwelling Mild Cognitive Impairment	Exercise (Tai Chi, aerobic, functional task, multi-component)	Varied	**Outcomes**: Cognition function (global cognition, executive and / or memory) **Outcome Measures**: **Global** (MMSE, ADAS-Cog, CDR-SOB, NCSE (Chinese version of Neurobehavioral Cognitive Status Examination), and MoCA). **Executive Function** (WAIS, WIAS-R, TMT, Verbal fluency test, Stroop colour Word Tests, Hopkins Verbal Learning test and digital span test).	**Global Cognition**: A positive effect was observed in 5 of these 9 studies. **Executive**: Four of these 7 studies (65%) found modest positive effects.

Carvalho 2014	Older adult ≥ 60 years; with or without mild cognitive impairment or disease. 27 (10 RCTs, 16 prospective cohorts, 1 case-control, 1 observational study). RCTs with ≥ 30 participants; follow up period of ≥ 6 months; all observational studies with ≥ 100 participants; published in English. : Studies with participants with systematic disorders such as COPD, diabetes, traumatic brain injury, or comorbidities that precluded participation in exercise programs.	Asia, USA, Europe	Older population of age ≥ 60 years	Mixed population (could not ascertain gender proportion of some studies); sedentary; independently ambulatory, living independently,	PA (resistance training, aerobic, strength, balance and flexibility; combination.	Flexi-tone (tri-weekly training of 10mins warm-up, 25-30 mins of strength, flexibility and balance, 10 min cool down); No intervention; Education to improve lifestyle and PA; Balance and tone training: stretching, range of motion, balance exercises, and relaxation technique; Social Interaction; one weekly training session consisting of warm-up and stretching exercises, but no overload training	**Outcomes:** Cognitive status / function, Brain Volume. **Outcome Measures:** Rivermead Behavioural Memory Test, Wechsler Adult Intelligence Scale, Direct and Indirect Digit Span, Memory Complaints Scale, Cambridge Cognitive Test, Wechsler Adult Intelligence Scale III, Wechsler Memory Scale-Revised, Toulouse-Pieron concentration attention test, Ray-Osterrieth complex figure, Freed and Cued Selective Reminding Test, Trail Making Test, and Stroop Test, MMSE, 3MS ADAS-Cog, Brain volume using MRI, Neuropsychological battery test, Reaction time tests including simple reaction time, 8-choice reaction time, 8-choice incompatible reaction time, and Go/No-Go reaction time Stroop Colour and Word Test, Wisconsin Card Sort Test, The Rey Auditory Verbal Learning Test	PA confers a protective effect on cognition in elderly subjects. 26/27 of all studies showed positive association between PA and cognition while 9/10 RCTs showed a positive association between PA and cognition.

Chang 2012	Healthy adults without cognitive impairment or specific disease, adults with an average age older than 65 years. 10 RCTs. Studies published in English; Studies in which cognitive performance was assessed by cognitive tasks or neuropsychological assessments; studies that included control or comparable groups.	NR	OAP mean age ≥ 65	Healthy adults without cognitive impairment or specific disease, adults with an average age older than 65 years	Resistance Exercise (Otago, resistance training and balance, ST with aquatic exercise, callisthenic training with aquatic exercise, Aerobic exercise and diet.	Health Education; Flexibility and Relaxation; balance and toning	**Outcomes:** Cognition. **Outcome measures**: WAIS-R, TMT-Word-list memory test, word-list recall test, verbal-fluency test, modified Boston naming test, constructional praxis & clock-drawing test B, Stroop CW, COWAT, WMS-R, Auditory oddball task, WAIS III, Toulouse-Pieron's concentration attention test, Mental arithmetic, computerized mirror drawing task, Rey-Osterrieth complex-figure test.	Designs including loads from 60% to 80% 1RM, approximately seven movements in two sets with 2 minutes rest between sets at least twice per week for 2–12 months (usually 6 months), might positively affect cognition in older adults

Coelho 2013	OP with a mean age above 60.0 years; healthy persons, subjects with a chronic disability or disease; subjects with a chronic disability or disease. 5 RCTS and 1 Non-RCT, Acute and training exercise protocols (low to high intensity); randomized controlled trials, randomized non-controlled trials and non-randomized controlled trials; and assessment of peripheral (serum and plasma) BDNF concentrations. Excluded articles that concerned animals; correlational studies and reviews; no exercise/training intervention; no assessment of peripheral BDNF.	NR	mean age = 66.2 years;	Older women (no-frail and pre-frail); mean age = 66.2 years; OP with MCI; elderly subjects with glucose tolerance criteria for pre-diabetes or newly diagnosed; patients with major depression and healthy OP.	Physical exercise (Resistance-training; Aerobic exercise (treadmill, stationary, bicycle, or elliptical trainer); Nordic walking; Gymnastics; Acute aerobic exercise (treadmill).	Stretching exercise; Unspecified; no control.	**Outcomes**: Peripheral serum and plasma BDNF (brain-derived neurotrophic factor) concentrations; Cognitive function; depression. **Outcome Measures:** Blood analysis (Plasma/ELISA, Serum/ELISA). MMSE, GDS, Spatial memory paradigm, Episodic memory performance (auditory verbal learning test), Symbol-digit modalities, verbal fluency, stroop, trails B, task switching, story recall, and list learning, HAMD (Hamilton Rating Scale for Depression), and Dem Test.	Aerobic exercise increases BDNF in older adults.

Colcombe 2003	Older adults (55-80 years). 18 randomized fitness intervention trials that included control groups and on fitness training that extended from several months to several years	NR	Young old (55-65), middle-old (66-70) and old-old (71+)	Community-dwelling, "normal" older adult; Sedentary. clinical populations of one kind or another, ranging from depressed persons to geriatric mental patients and individuals with cardiopulmonary obstructive disorder	Aerobic Fitness supervised aerobic training, combined aerobic training.	Any	**Outcomes:** Cognitive Function (Speed, visuospatial, controlled-processes and executive-control processes). **Outcome Measure**: MA of ES	Significant difference in overall ES (Overall ES for Intervention group was 0.478 (SE=0.029, n=101, P<.01), and Overall ES for control group was 0.164 (SE=0.028, n=96. P<.05). Aerobic fitness training improved cognitive performance in the older adults and markedly in the executive processing of the brain. The mid-old and old-old reported to have benefited more from fitness training compared with the young-old participants.

Gates 2013	Older adults (>= 65 years) with MCI either via clinical diagnosis of MCI on documented criteria or MMSE mean score of 24-28 inclusive. Study articles with intervention that is physical exercise training consistent with the definition of the American College of Sports Medicine. No limitations were imposed based on modality, dose, intensity, or supervision, but exercise had to be prescribed specifically. Studies of less than four weeks exercise did not meet criteria for training and were excluded. Diagnosed with physical or cardiovascular conditions preventing exercise; Other neurological conditions. Studies with any kind of control group including no contact, no treatment, waiting list, attention control, sham exercise, or alternative active treatment; With validated neuropsychological test of cognition reported at baseline and follow-up. 14 RCTs; Full-length article published in a peer-reviewed; English language journal	NR	65-95 years	predominantly female; With cognitive impairment; frail elderly	Physical Exercise (Isolated moderate intensity aerobic exercise, low intensity walking, resisted training, combined training, Tai Chi, supervised aerobic training, and combined aerobic training. Aerobic training make up half of studies).	Any (social visits, no contact, education programs, normal and recreational activities, sham, active control)	**Outcomes**: Cognitive function (Executive function, memory and information processing). **Outcome Measures**: MMSE, ADASCog, CAMCOG, WAIS-R. **MA of ES was performed.**	Overall, exercise training had minimal but positive effect on verbal fluency (ES=0.17; 95% CI= 0.04, 0.30). Aerobic training effective on global cognition in three studies [(ES=0.74; 95% CI: 0.43, 1.05), (ES=0.56; 95%CI: 0.19. 0.92), (ES=0.69; 95%CI: 0.03, 1. 32)]. Isolated resistance training produced significant effects on memory [(ES=3.37, 95%CI: 2.27, 4.74), (ES=0.54: 95%CI: 0.02, 1.08)]. Aerobic exercise training did not improve executive function compared with other reviews.

Kelly 2014	25 RCTs. Trials were included that investigated the effects of aerobic exercise, resistance training, or Tai Chi interventions on the cognitive function of community dwelling older adults (>50) with no known cognitive impairment.	International	Mean ages (65-84 years)	Community dwelling older adults	Aerobic, Resistance training and Tai Chi	Active and no intervention control groups	**Outcomes:** Cognitive function, divided into the domains of memory and executive function. Sub-categories were created within each domain. Memory domain sub-categories were: recognition, immediate recall, delayed recall, face-name recall, and paired associates. Executive function domain sub-categories were: working memory, verbal fluency, reasoning, attention, and processing speed. Composite measures of cognitive function were also included. Secondary outcomes of interest were subjective cognitive performance and activities of daily living (ADL).	**Aerobic exercise versus stretching/toning:** There were no significant differences between aerobic exercise versus stretching/toning on immediate recall (p = 0.62), delayed recall (p = 0.16), working memory (p = 0.30), attention (p = 0.15), or processing speed (p = 0.28). **Aerobic exercise versus no exercise active control**: control. Results from the meta-analysis revealed no significant differences between aerobic exercise and 'no exercise' active control groups on the measures of recognition (p = 0.51), immediate recall (p = 1.00), delayed recall (p = 0.67), verbal fluency (p = 0.58), reasoning (p = 0.28), working memory (p = 0.75), attention (p = 0.56), processing speed (p = 0.76), or cognitive function (p = 0.26). **Aerobic exercise versus no intervention:** Meta-analyses on available data revealed no significant differences between aerobic exercise and 'no intervention' control groups on measures of immediate recall (p = 0.30), delayed recall(p = 0.13), verbal fluency (p = 0.14), reasoning (p = 0.48), working memory (0.70), and processing speed (p = 0.17).

								**Resistance versus stretching/toning:** Three trials revealed significant improvements in performance for experimental versus control on measures of reasoning (p < 0.005) but not on measures of working memory (p = 0.47) or attention (0.37). Resistance versus no exercise active control: No significant differences on measures of working memory (p = 0.31) or attention (p = 0.62). **Tai Chi versus no exercise active control:** Meta-analysis on pooled data from two individual trials revealed significant differences between Tai Chi experimental groups and 'no exercise' control groups on measures of attention(p < 0.001) and processing speed (p < 0.00001), with differences for working memory approaching significance (p = 0.07).

Ohman 2014	Subjects with MCI or dementia. RCTs, physical exercise was the main intervention and cognitive function, assessed using neuropsychological or cognitive tests, was the outcome measure. 22 RCTs (8 MCI and 14 Dementia). Articles not written in English were excluded; other psychiatric, neurological disease, severe cardiac disease, impairment in ADL, use of donepezil / pharma-intervention.	NR	Age range: 50-86	60% female; mean MMSE score of 24	Physical exercise (aerobic exercise, strength training, balance, dual tasking, walking, hand and face exercises, TaiChi, treadmill, stationary bicycle, elliptical trainer	Social visits or normal social activities; educational material; stretching; health education.	**Outcomes**: Cognition; **Outcome Measures:** MMSE, ADAS-Cog, Symbol digit, verbal fluency, Stroop and task switching, delayed recall, CDR-SUB, Stroop Test, WMS-LM	There were some positive effects on one or several domains of cognition, global cognition, executive function or attention.

Paterson 2010	Population samples included asymptomatic "community-dwelling" older adults between 65 and 85 years of age. Restricted to participants with "minimal" initial impairment or functional inability. 34 (12 intervention studies)	International included 1 non OECD studies	NR	NR	Physical activity (strengthening , aerobic training)	NR	**Outcome**: Cognitive function (speed, visual memory, visual reproduction, verbal memory, motor function, working memory, executive function, cognitive inhibition and auditory attention. **Outcome Measure**: Varied (simple reaction time, choice reaction time, Wechsler Memory Scales, Benton visual retention test, Randt memory test, Wechsler memory scale, finger tapping, digit span tests, face recognition, verbal fluency, problem solving, word comparison, Stroop test, letter search, visual search, Digit span forward.	58% of intervention studies demonstrated small positive effects on at least one measure of cognitive function. These studies employed moderate intensity aerobic physical activity interventions; however, it is difficult to quantify the actual volume of exercise used in each intervention.
Sherder 2014	Older people (+55 years) with and without cognitive impairment. 8 (5 RCTs involving normal cognition (NC) and 3 with cognitive impairment	NR	Age ranges from 55-73 yeas for NC; 75-86 for participants with cognitive impairment.	NR	Walking	Flexibility, balance, strengthening, toning, social visits, no treatment	**Outcomes:** Executive Function; **Outcome Measures**: Spatial Word Memory, spatial switching, Trial Making Test, Stroop test, Verbal Fluency test, Digit span,.	Studies suggest that walking improved executive functions in cognitively intact older persons who have lived a sedentary life

Tseng 2011	Participants were older adults (age 65 years); The intervention involved a planned, structured, repetitive, and purposive exercise training or physical activity program; Outcomes included cognitive function; and type of trial was a randomized controlled trial (RCT). 12 RCTs.	NR	Mean age = 71.5 years	Older adult participants with and without cognitive impairment were 50%, respectively, and only 16.7% of the trials focused on female participants.	Physical Exercise (walking, treadmill running, extremity stretching exercise, weight bearing strength training, and swimming).	No treatment, Stretching, normal daily activities, educational materials, social visits, vitamin B supplements	**Outcomes:** Cognitive Function; **Outcome Measures**: MMSE, WAIS III WMS-R, ADAS-Cog, CERAD, CDR, SCWT, WCST, AVLT, VFT, DSST	Trials showed a positive effect for exercise on cognition when the exercise regimen lasted for 6 weeks and occurred at least three times per week for 60 minutes

Uffellen 2008	Cognitively healthy older adults or adults with cognitive decline or dementia but no mental disorders other than dementia, such as depression. (1) Design: randomized controlled trial; (2) intervention: physical exercise program; and (3) outcome: cognitive function assessed using neuropsychological tests. (4) Full-text articles written in English. 23 RCTs.	NR	>=55 years	Age of the study populations ranged from 55 to 94 years in cognitively healthy populations and from 67 to 99 years in populations with cognitive decline. In both groups, the majority of participants were women.	Physical exercise (Aerobic; Strength; Flexibility; Balance or a combination of the above.	Yoga, Exercise (strengthening, balance, flexibility)	**Outcomes:** Cognition function. **Outcome Measures**: WAIS, WIAS-R, TMT, Verbal fluency test, Stroop colour Word Tests, Visual reproduction, digit span; visual reproduction, verbal memory, verbal pairs test (mental status test (Strub and Black), based on WMS	This review suggests that different kinds of exercise may benefit cognitive function irrespective of baseline cognitive status. Five out of the 15 studies in cognitively healthy subjects observed significant beneficial effects on some of the included measures for cognition. Significant effects were also observed in 5 out of the 8 studies in subjects with cognitive decline. In cognitively healthy adults, improvements were observed in memory (Corsis block-tapping test, Rey – Osterrieth figure, face recognition, digit span) information processing abilities (organization, auditory processing), and executive function (word fluency). Effective interventions in this group included aerobic exercise (n = 2); strength exercise alone or combined with balance exercise (n = 1); and all-round exercise including aerobic, strength, balance, and flexibility training (n = 1).
