# Supplementary material for: Physical Activity in Community Dwelling Older People: A Systematic Review of Reviews of Interventions and Context
Source: PLoS One. 2016 Dec 20;11(12):e0168614. doi: 10.1371/journal.pone.0168614 (PMC5173028; doi:10.1371/journal.pone.0168614)
Supplement: S2 Table — (PDF) [file pone.0168614.s002.pdf]

S2 Table: Characteristics of included systematic reviews in question 2 (Interventions effective for increasing PA uptake and maintenance in 55+)

Supplementary Information

Study	Included Studies, Eligibility Criteria and Design	Country	Age	Population	Intervention	Control	Relevant Outcomes / Measures	Findings
Asikainen 2004	28 RCTs in total; RCTs with sufficient quality more than 25 participants and less than 35% of drop outs.	NR	50-65	Postmenopausal women aged 50 to 65 years; 2632 women in total. Healthy, sedentary or had some leisure PA at entry into study; **Special Population:** Women with diseases or risk factors such as dyslipidaemia, hypertension, obesity or osteoporosis	Nine studies used Walking; four studies used combined aerobic exercise (walking + flexibility+ one of cycling, swimming, dance); Nine studies used combined resistance training and aerobics; Two studies used resistance training with weight machines. Five other studies used other resistance training of five to nine exercises.	Not reported	**PA uptake and Maintenance**: Mean drop out, mean attendance. **Other Physiological and QoL measures relevant to PA uptake (Short term):** Health-related fitness (bodyweight; proportion of body fat of total bodyweight (F%); bone mineral density (BMD); bone mineral content (BMC); various tests on muscle performance, flexibility, balance and coordination; maxi-mal oxygen consumption (VO2max); resting blood pressure (BP); total cholesterol (TC); high-density lipoprotein-cholesterol; low-density lipoprotein-cholesterol; triglycerides; blood glucose and insulin). **Adverse Outcome:** Injury Rates	**PA uptake / Maintenance / AE: Walking** (mean drop out was 13%, mean attendance in four studies was 84%. Mean injury rate reported in six studies was 3%). **Combined Aerobics** (Mean dropout rate was 12%; Attendance rate reported in one study was 77% in home based exercise and 53% in group based exercise; Incidence of injury in this study varied from 23% (high intensity exercise and 13% for low intensity exercise). **Combined aerobics and resistance training** (Mean dropout rate was 15% and mean attendance was 67%. Mean attendance was higher in exercise groups with more aerobic component compared with resistance training; Mean injury rate was 6%). **Resistance training with weights** (mean dropout rate was 16% and mean attendance was 90%; Injury rate was 33%). **High impact resistance training** (mean attendance rate was 68%, Injury rate: 8%). **One single resistance, back extensor or jumping exercise** (mean attendance: 91%, injury rate: 2-53

Clegg 2012	6 RCTs in total; Studies in which the target population were selected on the basis of the presence of a specific medical condition. Studies conducted in care home facilities, were excluded. Studies in which the intervention included a mix of home-based and group-based exercise were only included if the home-based component formed the greater proportion of the intervention. Trials in which the intervention had been delivered as the main component of a falls prevention package were also excluded from this systematic review.	International (3 in Western Europe, 2 in USA and 1 in New Zealand)	The median age was 83 years (range 78– 88)	Frail older people; 987 participants. The majority of participants were female (median 79% female, range 50–88%). Three of the trails recruited less than 100 subjects; only two recruited more than 200 subjects. **Special Population**: Frail OAP; limited mobility requiring the use of a walking aid; sedentary; in receipt of home care and housebound but able to get out of chair and bed.	Home based exercise. One intervention included a single component of progressive resistance exercise. Two combined progressive resistance exercises with one or more additional components of flexibility, balance, walking or range of motion exercises. Two interventions were complex interventions combining multiple exercise components with an occupational intervention. One study used an electronic device that counted the number of sit-to-stands	NR	**PA uptake and Maintenance:** Completion and adherence rates **Other Physiological and QoL measures relevant to PA uptake (Short term):** Measures of mobility (TUG), HRQoL (EQ-5D) and ADL (Barthel Index), muscle strength, balance, depression, bone strength. **Adverse Outcomes:**	**PA uptake and Maintenance:** Median completion rate reported in six studies was 83% (65%-88%). Median adherence rate reported in three studies was 78% (66%-89%). **Other Physiological and QoL measures relevant to PA uptake (Short term):** One high-quality trial reported improved disability in those with moderate but not severe frailty. Meta-analysis of long-term care admission rates identified a trend towards reduced risk (pooled risk ratio, 0.89; 95% confidence interval, 0.55–1.45). Improved gait speed was reported in one trial, a trend towards improved gait speed was reported in one further trial, and gait speed did not improve in two. Improvements in ADL were reported in one trial; no improvements in ADL were reported in the other three trials. Three trials measured muscle strength using upper and lower body strength or grip strength. One trial reported improved lower body strength. There was no improvement in either upper or lower body strength in one trial. No improvement in grip strength was recorded in the study that measured this outcome. No improvement in general physical performance was reported in one trial. Improved balance was reported in one trial but there was no effect on balance in three trials. There was no effect on depression, bone density or flexibility.

Chase 2013	20 Intervention studies; Randomised Controlled studies that describe at least two groups; (a) were written in English, (b) published from 2000 to 2012, (c) described at least two-group study designs, (d) tested interventions designed to improve PA behaviour, (e) sampled community-dwelling adult subjects aged 60 years or older, (f) were regardless of health status, and (g) that measured PA behaviour as an outcome. Studies that did not include randomization or had samples consisting of 30 participants or less were excluded	International	Study age ranges were from 66.30 to 81.70 years.	Community-dwelling adult subjects aged 60 years or older. 3148. Sample size ranged from 33 to 966. Two studies had 100% female participants; one study had no female participants. Three studies did not report gender. In other studies female participants made up between 16.0% and 72.0% of the sample.	Five studies employed behavioural interventions only; Twelve studies combined cognitive and behavioural interventions.	Controls or usual care control groups	**PA uptake and Maintenance:** PASE, Pedometer and accelerometer, Modified 7-day activity interview, activity diary, Leisure-time PA questionnaire expressed in energy expenditure units, 7-day PA recall instrument, Baecke PA scale, Yale PA Survey, Auckland Heart Study PA questionnaire, Modified CHAMPS, Flemish PA Computerised questionnaire.	**PA uptake and Maintenance**: Two cognitive-based interventions using Motivational Interviewing strategies demonstrated success in significantly improving PA behaviour among participants. All studies using 'supervised exercise sessions alone' demonstrated non-significant findings in differences in PA behaviour between treatment and control groups at outcome. Inconsistent success in increasing PA behaviour was observed among studies combining Cognitive-Behavioural Interventions; however most studies using combination cognitive-behavioural interventions reported successful long-term findings. One cognitive-behavioural–based intervention demonstrated long-term PA behaviour change results, with evidence of continued higher levels of PA from baseline up to 2 years beyond the end of a study. There were no significant changes in PA outcomes from post-test to follow-up period in three studies (Kelly, 2004, Talbot, 2003, Bird, 2011).

Chase 2014	101 comparisons (48 single group comparisons and 53 two-grouped treatment versus control comparisons). Eligible primary studies (a) were published from 1960–2013; (b) tested PA interventions; (c) among community- dwelling older adults; (d) age 65 and older, or with a sample mean age of 70; (e) contained at least five participants; (f) reported enough data to calculate an ES; Published in English.	International	Mean age ranging from 68 - 88 years	community-dwelling older adults	Varied	NR	PA Uptake and Maintenance: Effect sizes (Cohen's d). Small (≤0.20), medium (=0.50), or large (≥0.80)	**PA Uptake and Maintenance**: PA interventions had a significant impact on PA behaviours among community-dwelling older adults. Overall mean ES for two-group post-test comparisons was 0.18 ($p < .001$). This ES is equivalent to a difference of 620 more steps per day or 73 more minutes of PA per week for the treatment group over the control group. The overall mean ES for two-group pre-post-test data studies was 0.17 ($p < .001$).The overall mean ES for studies designed as single group pre-post-test comparisons was 0.23 ($p < .001$). The overall mean ES for control group pre-post-test comparisons was 0.01 ($p = .78$). PA interventions tested among healthier subjects ($d = 0.30$) were more effective in improving PA behaviour than chronically ill subjects ($d = 0.11$) ($p = .03$). Interventions delivered through audio-visual media ($d = 0.48$) and mailed materials ($d = 0.34$) were more effective than without. Studies reporting theory-based interventions ($d = 0.28$) had larger effects than interventions without a stated theoretical basis ($d = 0.05$) ($p < .01$). Interventions employing combination cognitive and behavioural strategies were more effective ($d = 0.23$) than interventions using either strategy type alone ($d = 0.02$) ($p = .03$). Regarding specific intervention components, problem solving techniques and barriers management, appeared more effective in changing PA behaviour. The most common theories reported were the Transtheoretical Model and Social Cognitive Theories.

Conn 2003a	43 primary studies; studies that attempted to increase episodic exercise or overall physical activity among aging adults. The study contained a minimum of 5 participants. Data were reported during the years 1960 through 1999. The study examined overall physical activity (total amount of body movement) or episodic exercise behaviour (structured repetitive large muscle movement) as an outcome variable. The research report was in English.	NR	Mean participant ages in the primary studies ranged from 60 to 77.2 years.	Community-dwelling individuals 60 years or older. 33,000 aging adults	Unspecified in paper	NR	**PA uptake and Maintenance:** Overall PA and episodic exercise behaviour.	**PA uptake and Maintenance:** Interventions targeted to PA exclusively are more effective than those targeting multiple behaviours. Studies without health education were more effective than those reporting that they taught health benefits. MA findings strongly support the importance of some self-monitoring system to increase adults' PA. Meta-analysis revealed that the intensity of the intervention, in terms of contact time between the activity professionals and elders, is important. Intense direct contact with staff more than doubled the effect size of the interventions. In contrast, mailed or telephone interventions made no difference in outcomes. Although staff contact time is expensive, the profound effect on elders' physical activity behaviours makes this an important aspect of programming. Elders who exercised at centres as compared to home based activity, were much likely to continue PA. The most effective part of an activity prescription is making specific intensity recommendations. OAP were more likely to increase their PA when recommendation was for moderate intensity activity than when low intensity activity was suggested. Interventions delivered to groups were considerably more effective than that delivered to individuals. Interventions that recommend walking are probably more effective than programme without a walking suggestion. Walking is easily accessible and may be perceived as 'natural'.

Conn 2003b	17 RCTs; Minimum of five participants. Reported during the years 1960 through 2000. Directly measured overall physical activity or episodic endurance exercise outcome variables. Used a randomized, controlled trial design with statistical analysis comparing treatment and control groups. Research report in English.	NR	Mean subject age of 65 and older.	6,391 subjects.	Walking. Also 6/17 studies looked at overall PA	NR	**PA uptake and Maintenance:** Overall PA and episodic exercise behaviour (<= 6 months post-test). Exercise maintenance (> 6 months post-test)	**PA uptake and Maintenance:** 7/10 studies with theory based intervention reported positive findings. 5/7 studies which used social cognitive framework reported positive results. 2/3 studies that used TTM reported positive outcomes. 4/5 that used combined models reported significant treatment effects. 3/5 studies with supervised centre based exercise reported positive treatment effect. 7/12 studies without supervised exercise reported greater exercise in treatment group than in the control group. Further, 4/6 studies with individualised interventions reported greater exercise in the treatment groups than the control groups. Interventions delivered to individuals were about equally likely to result in positive (6/11) and negative findings (5/11). Non- PA intervention used included motivational strategies and behavioural change techniques such as self-regulation, social support, stimulus control, self-regulation, health education etc. Self-monitoring and health education were most commonly used in studies. Mixed results on the association between these BCTs and PA uptake. 3/5 studies that delivered interventions in subjects' homes reported positive results. Each of the four studies that delivered interventions in aggregate community sites such as senior centres or churches reported more exercise by experimental subjects than control. **Maintenance:** 5/17 studies with >=6 months follow-up reported significance maintenance of PA after end of intervention period. 2/17 reported no difference between treatment and control.

Cyarto 2004	8 intervention (RCT) studies including one review containing 21 trials; Include only intervention studies with participants aged 60 years or older;	International (4 USA, 1 Australia, 2 UK, 1 Belgium)	The age of participants ranged from 40 to over 90 years, with approximately even representation of the 'young' old (mean age 50-60) the 'mid old' (mean age 60-70), and the 'older old' (mean age over 70)	Participants aged 60+. Sample sizes ranged from small studies with only 17 and 20 participants, to one with 719. Most studies had between 75 and 300 participants.	Progressive Resistance Training (Walking, strength training, flexibility, balance and co-ordination)	NR	**PA uptake and Maintenance:** Questionnaires measuring PA, exercise logs. **Other Physiological and QoL measures relevant to PA uptake (Short term):** Accelerometers, heart rate monitors, measurement of CV-risk factors and direct observation of participants.	**PA uptake and Maintenance:** All the general practice interventions produced some positive impact on PA levels. Study that used community mass media communication reported 23% increase in walking and proportion of those achieving 30 minutes of activity a day. Most of the studies reported positive results. **Maintenance:** CHAMPS II study reported maintenance of FU after 12 months. A notable feature of this study was the long-term involvement of the local community in the project through a local advisory committee.
de Vries 2012	18 original studies (21 papers) included in the review. The study design was a randomized controlled trial. The included patients had to be physically frail as defined by the study authors using specific criteria on the presence of mobility problems and/or physical disability and/or multi-morbidity. The included patients had to be older adults aged ≥60 years old, living in the community. Interventions should consist of physical exercise therapy defined as exercises or a combination of exercises aimed at improving the already decreased levels of mobility, strength,	NR	The age of the study population varied from 60 to 85 years.	Community-dwelling older adults with impaired mobility, physical disability and/or multi-morbidity. **Special Population:** Frail and with mobility problems	Physical exercise therapy (Strength training, balance training, functional and task-related training, endurance and mobility training	No exercise, low-intensity exercise.	**PA uptake and Maintenance:** PASE, self-reported PA, YPAS, FAI, number of walks. **Other Physiological and QoL measures relevant to PA uptake (Short term):** WS timed chair stands, SF-36, IADL, TUG, 6MWT, 400MWT.	**PA uptake and Maintenance:** Three studies evaluated their intervention on the level of physical activity. Two of these studies were pooled in a meta-analyses, which showed no exercise on PA level (SMD: 0.08, 95% CI: -0.21, 0.31, I2: 0%) None of these studies found a significant effect. However, results show that physical exercise therapy has a positive effect on mobility.

	endurance, balance and/or physical activity. The study had to use one or more of the following outcomes: mobility, physical functioning, and level of physical activity and/or quality of life.							
Fairhall 2011	15 trials included in MA. specified participants were aged 60 years or over, clearly recruited participants described as elderly or senior, or in the case of trials including younger participants with a specific diagnosis (e.g. stroke), had a mean age minus one standard deviation exceeding 60 years	International	Aged 60+	3,616 participants	Exercise interventions that aimed to reduce falls in older people (Strength, balance, Tai Chi)	The effects of the intervention were compared with placebo, alternate therapy or usual care.	**PA uptake and Maintenance:** Participation in life role measured by Scales with ICF components such as Adelaide Activity Profile, PASE, Older American's Resources and Services, Nottingham Extended ADL Index, Lawton's IADL Scale, Late Life Function and Disability Index, The Groningen Activity Restriction Scale, Frenchay Activities Index, Falls Handicap Inventory.	**PA uptake and Maintenance:** The pooled estimate of the effect of interventions including exercise indicated a small improvement in participation (Hedges' g = 0.16, 95% confidence interval = 0.04–0.27, P = 0.006). Meta-regression showed multifactorial intervention with an exercise component had a larger effect than exercise intervention alone, but the difference was not statistically significant (effect on Hedges' g = 0.22, 95% CI = −0.05 to 0.50, P = 0.10).

French 2014	25 Intervention studies in total were included (16 for PA). Eligible studies were required to include community-dwelling samples of older adults (mean age ≥60 years old) that were not defined by a clinical condition. Eligible studies were those reporting on a change in self-efficacy following an intervention to increase frequency or duration of lifestyle or recreational physical activity.	NR	The overall mean age of participants was 69 years (study means ranged from 60 to 84 years)	Community-dwelling adults 60 years or over. The mean number of participants in the comparisons included in the self-efficacy analysis was 247 (range 5 to 1,011); the mean number included in the physical activity analysis was 349	Lifestyle PA (gardening, walking); Exercise (Aerobic class, gym, jogging), others	NR	**PA Uptake and Maintenance:** Change in Physical Activity measured in 'd' Cohen ES	**PA Uptake and Maintenance:** (Pooled ES) BCT Interventions had a small effect on PA (d=0.14, 95% CI 0.09, 0.2, p<0.001). Effect size ranged from d= -0.02 to 0.63. **(Individual results)** 3 BCTs were significantly associated with higher PA behaviour: ‘barrier identification / problem solving’, ‘provide rewards contingent on successful behaviour’, ‘model / demonstrate behaviour’. 10 BCTs were associated with lower PA behaviour. The greatest difference in effect size occurred when the following BCTs were present: 'provide normative information about others' behaviour', 'provide information on where and when to perform behaviour' and 'plan social support / social change'.
Geraedts 2013	Twenty-four studies met the inclusion criteria for systematic effectiveness evaluation and 22 for adherence inventory. (1) The study assesses a physical activity intervention program in the home situation. (2) The study includes at least one study group that receives the intervention exclusively in the home situation. (3) The study mentions remote feedback used in the physical activity program, which does not include any structural contact that is not remote except for effect measurements and explanation of or initiation into the exercise program. (4) The study addresses at least one aspect of general physical activity behaviour or	International	55+	5328 participants aged 55+	Remote feedback on Home based PA (Phone, Text)	Exercise or Non-Exercise	**PA Uptake and Maintenance:** Walking speed, Strength, balance, Peak VO2, 7-day PA recall, accelerometer, 6 MWT, TUG, adherence rate, compliance rate.	**PA Uptake and Maintenance:** Results show that PA programs with frequent and non-frequent remote feedbacks are equally as effective in enhancing physical capacity measures as supervised exercise without remote feedback. **Maintenance:** Adherence to interventions using remote feedback was higher in the control groups in studies where intervention groups were compared to TAU. Adherence to interventions using remote feedback seems mostly acceptable-to-good, with rates in intervention groups varying between 32.1 and 91%.Oe study compared text messaging to a phone strategy and found that texting led to a significantly higher adherence than phone.

	physical capacity as a primary or secondary outcome measure. (5) The study concerns at least one group of participants aged 55 years and older on average. (6) The study is neither a case study nor a review (7) The article is in the English, Dutch or German language.							
Hobbs 2013	Included 21 trials, which assessed PA behaviour using objective or self-report measures; compared to a no-intervention, minimal or usual care intervention; or a different type of intervention; studied healthy participants or those 'at risk' of chronic disease with a mean or median age of 55 to 70 years. Publications of any language with an English language abstract and with a country of origin of one of the 'most developed countries' within the United Nations index	International (USA, Europe, NZ, Japan, Australia, Canada)	The mean age of participants was 60.7 years (SD = 4.4; range 55 to 67.6).	10519 at risk adults aged 55 to 70 years. 'At risk' participants were reported as having at least one of the following disease risk factors: hypertension, impaired glucose tolerance, overweight/obese, hyperlipidaemia, dyslipidaemia, family history, metabolic syndrome or osteopenia.	The majority of interventions were Multimodal and provided physical activity and lifestyle counselling.	Received usual care; exercise alterative; some studies included information leaflet and newsletters	**PA Uptake and Maintenance:** Five trials used pedometers deriving step-count and one trial used an accelerometer deriving vector magnitude. Twenty trials estimated PA duration by self-report questionnaires reported as minutes of PA or energy expenditure. Four trials assessed PA using both objective and self-report methods.	**PA Uptake and Maintenance:** Interventions to promote physical activity were effective at 12 months (standardized mean difference (SMD) = 1.08, 95% confidence interval (CI) = 0.16 to 1.99, pedometer step-count, approximating to an increase of 2,197 steps per day; SMD = 0.19, 95% CI = 0.10 to 0.28, self-reported physical activity duration outcome), but not at 24 months based on a small subset of trials. Further analysis by O'Brien (2015) shows that increasing the number of BCTs in PA promoting intervention does not enhance long term effectiveness. Interventions aiming to promote PA should consider using BCT feedback in order to enhance effects.
Muller 2014	17 intervention-studies. a) Study sample consisted of healthy, community dwelling adults aged 50 years or older b) Study implemented a non-face to-face intervention to initiate, increase and/or maintain PA, exercise and/or walking, c)	International (USA, Netherland, Australia, New Zealand	50+	Healthy, community dwelling older adults (≥ 50 years)	Non-face to face PA	NR	**PA Uptake and Maintenance:** Self-reported questionnaires / instruments, accelerometer, weekly time spent in PA, weekly energy expenditure.	**PA Uptake and Maintenance:** Of the 16 studies, 14 reported significant improvements in PA over the respective study periods (1 week to 24 months). Only one reported a non-significant decrease of PA in terms of daily calorie expenditure and time spent in moderate or greater PA over the previous week. **Maintenance**: PA levels were maintained after the intervention stimulus was removed in all but one study

	Quantitative data was used to report the effectiveness of interventions.							
Neidrick 2012	11 studies (8 RCTs, 2 non-RCTs and 1 qualitative study).the study assessed the effective- ness of physical activity promotion interventions implemented within the confines of a primary care setting; the aim of the study was to assess the efficacy of intervention(s) to increase levels of physical activity of older adults; and subjects were at least 50 years of age or older.	International (USA, Australia, Canada, Europe, England)	50+	NR	Standardized Intervention to promote PA	NR	**PA Uptake and Maintenance:** Self-reported questionnaires / instruments, PASE, Physical Activity Recall (PAR), Active Australia Physical Activity Questionnaire, Dutch Short Questionnaire to Assess Health Enhancing Physical Activity (SQUASH), Pedometer.	**PA Uptake and Maintenance:** 7/10 studies found that PA promotion intervention was effective in increasing PA. 1/10 found that generalised health behaviour modification without PA component was not effective. 1/10 studies found that supplementing verbal advice with written advice did not show a significant effect on PA uptake. **Maintenance**: Limited evidence to show effect on long term adherence.
Nigg 2012	18 RCTs in total (14 studies on PA or exercise behaviour). Included RCTs with PA, nutrition, tobacco, and alcohol behaviours interventions either individually or in some combination on older adults published in English language. The health behaviours included both adoption and cessation behaviours. Focus was on papers from 2006-2011. Studies with older adults (55+) from community and clinical settings were included.	NR	55+	NR	Single Health Behaviour Change Interventions (SHBC) and Multiple Health Behaviour Change Interventions (MHBC)	NR	**PA Uptake and Maintenance:** Self-reported questionnaires / instruments, accelerometer, weekly time spent in PA, weekly energy expenditure.	**PA Uptake and Maintenance:** Of the 12 SHBC studies evaluating PA or exercise, participants generally improved their level of activity at FU (6-12 months). MHBC showed mixed results; one study found that the combination of PA and fruit and vegetable consumption improved only nutritional outcomes but not PA behaviour at FU. The other showed improvement in both PA and Weight loss behaviour.

Stevens 2014	6 RCTs in total (5 RCTs and 1 Cluster RCT); Tailored physical activity interventions including aerobic, strength and balance exercises that recruited participants (aged 50 and over) from and/or were provided in general practice. 'Tailoring' in this review means baseline assessment of current physical activity and functional limitations, and individualised recommendations to increase physical activity.	International (USA, NZ, Australia, Canada, UK)	Five of the studies reported the mean age of participants, which ranged from 65 to 74; four recruited a greater number of females	Adults aged 50 and above	Tailored PA (aerobic, strength and balance exercises)	NR	**PA Uptake and Maintenance:** PASE; Time to reach target of >= 90 mins / week of MVPA; Auckland Heart Study Exercise Questionnaire; Frequency and duration of walking and vigorous exercise.	**PA Uptake and Maintenance:** 2/6 studies reported a statistically significant increase in physical activity levels. Two studies showed no significant increase in activity.

Van der Bijj 2002	38 RCTs in total. Included if RCTs design, average sample population age of >=50 years and minimum age of 40 years; intervention consisting of an exercise program or aimed at promoting PA otherwise (e.g., information and counselling); and information on participation or PA levels.	International (USA, Europe)	Mean age ranging from 51-88 years	Community dwelling, healthy and inactive older adults. Large majority were white, well-educated and had moderate to high incomes	Home based PA, Group based PA and Educational PA	NR	**PA Uptake and Maintenance:** Participation rates.	**PA Uptake and Maintenance: Home based PA** (Participation rate 86%-93%). Participation in longer term interventions was lower than in short-term Rx. 2 studies reported a decline in PA level after end of intervention. **Group based PA** (Mean participation in short term duration Rx (< 1 year) = 84%, 55-100%). Interventions in nursing or residential homes achieved high participation rates (mean= 87%). Participation rate in Rx > 1 year ranged between 63-84% mean=75%). All studies reported higher PA levels than baseline and that PA levels in intervention groups were significantly higher than in control. 1/3 studies achieved higher PA level at 10 year follow-up. **Education PA** (35%-96%); Participation rate declined with increase in intervention sessions. All educational interventions showed significant increase in PA compared to control in short term (< 1 year) duration. Overall high participation rates are achievable with short term PA interventions (< 1 year). However, these high participation rates were not found for long-term intervention. However, this relationship appears to be less strong in group-based compares to home based Rx. Possible explanation for inverse relationship between the participation rate and length of intervention are lack of interest, motivation, enjoyment, time or perceived benefit. It appears that group-based and education interventions were effective in increasing PA in short-term. Long-term education was ineffective. Insufficient data to show long-term effectiveness of group based interventions. Contrary to previous research, which shows that home based interventions were superior to group based in terms of PA promotion, participation rates appear to be comparable.
