# Supplementary material for: Physical Activity in Community Dwelling Older People: A Systematic Review of Reviews of Interventions and Context
Source: PLoS One. 2016 Dec 20;11(12):e0168614. doi: 10.1371/journal.pone.0168614 (PMC5173028; doi:10.1371/journal.pone.0168614)
Supplement: S3 Table — (PDF) [file pone.0168614.s003.pdf]

S3 Table: Characteristics of included systematic reviews in question 3 (Barriers and facilitators to PA in 55+)

Study	Included Studies, Eligibility Criteria and Design	Country	Age	Population	PA type	Barriers	Facilitators	Limitations
Barnett 2012	Studies were included if they explored experiences of or views on free-living PA around the transition to old age retirement in community-dwelling individuals, were published after January 1980 and used established qualitative research methods. 5 studies	International	NR	All participants had been retired for between six months and 5.6 years	recreational PA	Lack of time for recreational PA; Low perceived value of recreational PA and preference for productive / meaningful PA.	Health properties of PA motivate adoption/increase of recreational PA but do not guarantee long-term maintenance; Lifelong PA habits influence recreational PA patterns after retirement; Recreational PA provides a new daily routine; Recreational PA offers new personal challenges; Recreational PA provides opportunities for social interactions.	The qualitative evidence is limited by the small number of studies available and the limited socioeconomic diversity of study participants, who were mostly from relatively affluent backgrounds
Boehman 2013	Rural and remote older Australians. 5 studies	USA	50+	Community dwelling – people living independently in their home and not an aged care facility	Population-based falls prevention exercise programs	**(Personal)** Health, Lack of motivation, fatigue, time factors, lack of knowledge about exercise, low self-efficacy, feelings and perception about exercise, previous exercise experience, body image, fear. **(Social)** Lack of social support, family and household commitments. **(Environmental)** Poor built environment, lack of access to programs and facilities, safety concerns, dogs, traffic, weather, lack of transportation, costs.	**(Personal)** Health, Enjoying the activity, self-motivation, body image, previous exercise experience, exercise, exercise knowledge. **(Social)** Social support, social contact, recommendation to exercise, role models. **(Environmental)** Accessible facilities and programs, available transport, conducive built environment, low/ reasonable costs.	There were only five articles that focused on rural locations, all of which were from the United States, four focused on women only and the total population was 326 people.

Bunn 2008	The studies of interest were those that evaluated interventions to promote adherence to, or participation in, a falls-prevention programme or strategy, and that identified the factors that influenced whether older people participated and were compliant. 24 studies (mixed RCT, surveys, cross-sectional and qualitative studies)	International	55+	Older population	Falls prevention programme	**(General)** Fatalism/attributing falls to external causes/lack of knowledge about effectiveness of falls prevention; Perception that physical deterioration inevitable with age; Lack of relevant information in appropriate formats/ language; Provision of 'one size fits all ' advice. Advice seen as common sense/patronising; Low self-efficacy. Fear of loss of independence/risk taking ability; No perception of need for help (no previous falls); Provoking fear of falling by using scare tactics; Social stigma: association with old age/frailty; Differing agenda of older people and health professionals. (**Exercise)** No previous exercise 'habit'; Physical discomfort/unpleasant sensations associated with exercise; Underlying beliefs about personality type (e.g. too lazy, no willpower); Self-perception: too old to exercise. **(Home modifications / assistive devices)** Dislike of interventions seen as intrusive/didactic; Stigma of devices associated with old age.	**(General)** Information that falls can be preventable; Communicating life-enhancing aspects of strategies, e.g. maintaining independence and control; Accessible, appealing information format, from a variety of sources and in different languages; Choice of interventions for different people and lifestyles; High self-efficacy; Personalised modifications; Emphasis on social aspects of interventions. **(Exercise)** Previous exercise 'habit', Making exercise fun/enjoyable/ sociable, Good leadership/facilitation, Motivation/information about physical and psychological benefits of exercise, Programmes tailored to needs or lifestyle, Convenient scheduling/ reasonable pricing/good access and transport. **(Home modifications / assistive devices)** Facilitate feeling of ownership of interventions, shared decision making, Referral from health-care professional (especially doctor)	Potential for publication bias to affect results due to non-RCTs in review. Study quality not used to weight or exclude studies may also affect results

Child 2012	Studies were included if they examined influences on the implementation of fall-prevention programmes among community-dwelling older adults and used recognised qualitative methods of data collection and analysis. (12 studies reviewed experiences of community dwelling OA)	International	OA	community-dwelling older adults	Falls prevention programme	**(Practical consideration)** Cost of accessing programme; ease of access to falls-prevention intervention i.e. ability to drive, availability and cost of transport, car parking facilities, cold weather; Time **(Adapting for community)** social and cultural acceptability of assistive devices, types of exercise, and fatalistic attitudes towards falling **(Psychosocial)** transforming identity (independence, confidence and QoL)		
Cunningham 2004	The primary inclusion criterion was the relation between the built environment of neighbourhoods and the physical activity in seniors. 27 articles in total, 6 focused on seniors.	NR	Seniors	Community dwelling seniors	PA	Safety of footpaths, Access and convenience of facilities, proximity to services, heavy traffic, safety (dogs, crime), noise, adequate lighting, public transportation, litter.		Inconsistent findings and mixed results in primary studies of the relationship between PA and environmental factors.
Devereux-Fitzgerald 2016	14 Qualitative or mixed methods studies with a qualitative component reporting experiences of any intervention to increase physical activity whether in a randomised control trial or not; (b) all participants 65 years or older in line with the World Health Organisation's definition of older adult in relation to physical activity (WHO, 2010a); (c) all participants independently dwelling in the community, (d) paper written in English. Papers were excluded if:	USA, Australia, Canada, UK, Chile	65+	Independent community dwelling older people	PA	Self-Perception<br>Expectations<br>Doubts/Fears<br>Priorities<br>Passivity<br>Realistic Expectations<br>Measure Against Self<br>Objective Feedback<br>Choice/Flexibility<br>Accessibility<br>Continued Social Interaction	Making Friends<br>Social Support<br>Social Modelling<br>Commitment<br>Making a Contribution<br>Knowledge/Attitude of Trainer/Provider<br>One-to-One Attention<br>Accountability<br>Go at Own Pace	Vague methodological information; Limited SES data; limited information on BCTs used.

	(a) There was no physical activity intervention; (b) the physical activity intervention was for condition specific prevention, rehabilitation or disease management; (c) there was no qualitative report of experiences of intervention.							
Dunsky 2012	To be selected for this review, a study had to report the rate of injuries that occurred during physical activity or sport.	International	45+	Adults and older adults	PA and sports	Injury		Main finding was that the information on rate of injuries in purposeful physical and sports activities in advanced age was too limited for drawing a reliable conclusion. While there is some information regarding the rate of injuries, it is not presented relative to the extent of activities, either in terms of the number of active people or in terms of the intensity of the activity.

Franco 2015	132 studies involving 5987 participants were included	24 countries with most conducted in USA, UK and Canada	Mean age (60-89)	Community dwelling (85%); long-term care facilities, assisted-living facilities and hospitals.	Structured exercise programmes, other forms of physical activity or combination of both.	Social awkwardness<br>Encouragement from others<br>Dependence on professional instruction<br>Pain or discomfort<br>Concerns about falling<br>Comorbidities<br>Environmental barriers<br>Affordability<br>Apathy<br>Irrelevance and inefficacy	Valuing interaction with peers<br>Self-confidence<br>Independence<br>Perceived health and mental health benefits<br>Previous PA habits	Studies from developed countries

Horne 2012	Primary inclusion criteria were qualitative research with a SA sample (first, second or third generation), that discussed some aspect of exercise or PA (e.g. attitudes, beliefs), with average age of the sample ≥60 years. 10 studies	International	60+	OA from South Asian Community	PA	**(Communication)** Obtaining accurate information; Lack of information; Lack of support and encouragement; Language barriers. **(Relationship)** Overprotective family; Dependence on social support; Group norms. **(Beliefs)** Concepts of ageing; Lack of knowledge and understanding about the benefits of exercise and keeping active; Unfamiliarity of gym based exercise; Role of fate and lack of personal control. **(Environment)** Migration; Not socialised to spend time outdoors or doing sport; Lack of culturally sensitive facilities; Lack of knowledge of geographical area and facilities; Obligations to others and contribution to community activities.	**(Communication)** Who provides advice; Positive reinforcement; (**Relationship)** Facilitative relatives; Group, peer and community support; Instructor support. **(Beliefs)** Collectivist norms; (**Environment)** Engaging in community activities; Walking outdoors was a preferable form of PA.	
