# Supplementary material for: Physical Activity in Community Dwelling Older People: A Systematic Review of Reviews of Interventions and Context
Source: PLoS One. 2016 Dec 20;11(12):e0168614. doi: 10.1371/journal.pone.0168614 (PMC5173028; doi:10.1371/journal.pone.0168614)
Supplement: S4 Table — (PDF) [file pone.0168614.s004.pdf]

S4 Table: Quality assessment of SR on PA effect on cognition in older adults

AMSTAR (High= 8-11, Good= 5-7, Low= <5)

Key: 1. ‘a priori design; 2. duplicate study selection and data extraction; 3. comprehensive literature search; 4. Status of publication as an inclusion criterion; 5. List of studies (included and excluded provided)?; 6. Characteristics of the included studies provided? 7. Scientific quality of the included studies assessed and documented; 8. Scientific quality of the included studies considered in formulating conclusions; 9. Appropriate method to combine findings; 10. Publication bias; 11. Conflict of interest

Author (Year)	1	2	3	4	5	6	7	8	9	10	11	Ranking
Angevaren 2008	Y	Y	Y	N	Y	Y	Y	Y	Y	N	N	High
Balsamo 2013	N	N	Y	N	N	Y	N	N	N	N	Y	Low
Cai 2015	Y	N	Y	N	Y	Y	N	Y	N	N	N	Good
Carvalho 2014	Y	Y	Y	N	N	Y	Y	Y	Y	N	Y	High
Chang 2012	Y	U	Y	N	N	Y	U	Y	N	N	N	Low
Coelho 2013	Y	N	Y	N	N	Y	N	Y	Y	Y	Y	Good
Colcombe 2003	Y	N	Y	Y	N	N	N	Y	Y	N	Y	Good
Gates 2013	Y	Y	Y	N	N	Y	Y	Y	Y	N	Y	High
Kelly 2014	Y	Y	Y	Y	Y	Y	N	Y	Y	N	Y	High
Ohman 2014	Y	Y	Y	N	N	Y	Y	Y	Y	N	Y	High
Patterson 2010	Y	Y	Y	N	N	N	Y	Y	N	N	Y	Good
Sherder 2014	Y	Y	Y	N	N	Y	N	Y	Y	Y	Y	High
Tseng 2011	Y	Y	Y	Y	Y	Y	Y	Y	N	N	N	High
Uffellen 2008	Y	Y	Y	N	Y	Y	Y	Y	Y	N	Y	High
