# Supplementary material for: Physical Activity in Community Dwelling Older People: A Systematic Review of Reviews of Interventions and Context
Source: PLoS One. 2016 Dec 20;11(12):e0168614. doi: 10.1371/journal.pone.0168614 (PMC5173028; doi:10.1371/journal.pone.0168614)
Supplement: S5 Table — (PDF) [file pone.0168614.s005.pdf]

S5 Table: Quality assessment of SRs on interventions to promote uptake of PA in older adults

AMSTAR (High= 8-11, Good= 5-7, Low= <5)

Key: 1. 'a priori design; 2. duplicate study selection and data extraction; 3. comprehensive literature search; 4. Status of publication as an inclusion criterion; 5. List of studies (included and excluded provided)?; 6. Characteristics of the included studies provided? 7. Scientific quality of the included studies assessed and documented; 8. Scientific quality of the included studies considered in formulating conclusions; 9. Appropriate method to combine findings; 10. Publication bias; 11. Conflict of interest

Author (Year)	1	2	3	4	5	6	7	8	9	10	11	Ranking
Asikainen 2004	Y	U	Y	N	Y	Y	Y	Y	Y	N	Y	High
Clegg 2012	Y	Y	Y	N	N	Y	Y	Y	Y	N	Y	High
Chase 2013	Y	N	Y	N	N	Y	N	N	Y	N	Y	Good
Chase 2014	Y	U	Y	Y	N	Y	N	Y	Y	Y	Y	High
Con 2003a	Y	Y	Y	Y	N	Y	N	N	Y	N	N	Good
Con 2003b	Y	Y	Y	Y	N	Y	U	U	Y	N	N	Good
Cyarto 2004	Y	N	Y	N	N	N	N	U	Y	N	Y	Low
de Vries 2012	Y	Y	Y	N	N	Y	Y	Y	Y	N	Y	High
Fairhall 2011	Y	Y	Y	Y	Y	Y	Y	Y	Y	Y	Y	High
French 2014	Y	Y	Y	N	N	N	N	N	Y	N	Y	Good
Geraedts 2013	Y	Y	Y	N	N	Y	Y	Y	Y	N	Y	High
Hobbs 2013	Y	Y	Y	Y	N	N	Y	Y	Y	Y	Y	High
Muller 2014	Y	Y	Y	N	N	Y	Y	Y	Y	N	Y	High
Neidrick 2012	Y	Y	Y	N	N	Y	N	N	Y	N	N	Good
Nigg 2012	Y	Y	Y	N	N	Y	U	U	Y	N	Y	Good
Stevens 2014	Y	Y	Y	N	N	Y	Y	Y	Y	N	Y	High
Van der Bijj 2002	Y	Y	Y	N	N	Y	N	N	Y	N	Y	Good
