# Supplementary material for: Physical Activity in Community Dwelling Older People: A Systematic Review of Reviews of Interventions and Context
Source: PLoS One. 2016 Dec 20;11(12):e0168614. doi: 10.1371/journal.pone.0168614 (PMC5173028; doi:10.1371/journal.pone.0168614)
Supplement: S1 Appendix — (PDF) [file pone.0168614.s006.pdf]

S1 Appendix: Electronic Search Medline

Older people reviews 1, 2, 3: MEDLINE SEARCH STRATEGY FOR SRs (using BMJ SR filter and older-midlife search terms).

1. Health Behavior/

2. exp Risk Reduction Behavior/

3. exp Health Promotion/

4. exp Primary Prevention/

5. exp Preventive Medicine/

6. ((health$ adj3 behavior$) or behaviour$).ab,ti.

7. ((behavio?r$ or lifestyle or "life style") adj3 (change$ or changing or modification or modify or modifying or therapy or therapies or program$ or intervention$ or counsel$)).ab,ti.

8. (prevent$ adj3 (behavior or behaviour)).ab,ti.

9. ("health check" or "check up" or "check-up").ab,ti.

10. "health MOT".ab,ti.

11. "NHS check".ab,ti.

12. or/1-11

13. Diet/

14. *Food/

15. (diet or diets or dietary).ab,ti.

16. (dietary adj3 fat$).ab,ti.

17. salt$.ab,ti.

18. sugar$.ab,ti.

19. fruit$.ab,ti.

20. vegetable$.ab,ti.

21. (wholegrain or whole-grain or "whole grain" or "glyc?emic index").ab,ti.

22. (fish or "omega-3" or "omega-6" or "omega 3" or "omega 6" or (fish adj2 oil$)).ab,ti.

23. ("five a day" or "5 a day").ab,ti.

24. (fat$ adj3 (intake$ or diet$ or consum$)).ab,ti.

25. ((protein or carbohydrate$ or fibre or fiber) adj2 (intake$ or consum$ or diet$)).ab,ti.

26. ((energy or calorie$) adj2 (intake$ or consum$ or diet$)).ab,ti.

27. (vitamin$ adj3 (intake$ or consum$ or diet$)).ab,ti.

28. ((micronutrient or micro-nutrient or "micro nutrient") adj3 (intake$ or consum$ or diet$)).ab,ti.

29. nutrition.ab,ti.

30. *Food Habits/

31. *Food Preferences/

32. exp Nutrition Therapy/

33. or/13-32

34. eat$.ab,ti.

35. (over eat or "over eat" or overeat).ab,ti.

36. *Malnutrition/

37. malnutrition.ab,ti.

38. (undernutrition$ or undernourish$ or under-nutrition$ or under-nourish$).ab,ti.

39. (weight adj2 (gain$ or loss$ or cycling or reduc$ or maint$ or decrease$ or increas$ or watch$ or control$ or change$)).ab,ti.

40. ((bmi or "body mass index") adj2 (gain$ or los$ or cycling or reduc$ or maint$ or decrease$ or increas$ or watch$ or control$ or changes$)).ab,ti.

41. (obesity adj2 "related behaviour").ab,ti.

42. or/34-41

43. Exercise/

44. Sports/

45. *Exercise Therapy/

46. exp Physical Exertion/

47. exp "Physical Education and Training"/ or exp Physical Fitness/

48. exp running/ or exp swimming/ or exp walking/

49. exp Bicycling/

50. exp Dancing/

51. "tai chi".ab,ti.

52. tai ji/ or yoga/

53. "tai ji".ab,ti.

54. yoga.ab,ti.

55. ((center$ or centre$ or program$ or site$ or setting$ or venue$ or event$) adj3 (sport$ or exercise$ or fitness or training$ or activ$ or leisure)).ab,ti.

56. Life Style/

57. exp Sedentary Lifestyle/

58. sedentary.ab,ti.

59. (exercis$ or sport$ or danc$ or run$ or walk$ or jog$ or garden$ or leisure or recreation$ or golf$ or tennis$ or badminton$ or bowl$ or curl$).ab,ti.

60. (bicycl$ or training or trainer$ or bik$ or wellness).ab,ti.

61. balanc$.ab,ti.

62. ((resistance or conditioning) adj2 training).ab,ti.

63. ((cardio$ or aerobic$) adj2 (sport$ or exercise$ or fitness or training$ or activ$)).ab,ti.

64. or/43-63

65. Smoking/

66. exp Smoking Cessation/

67. exp "Tobacco Use Cessation Products"/

68. (smok$ adj3 (cessation or cease$ or quit$ or stop$ or reduce$ or reduction)).ab,ti.

69. or/65-67

70. exp Drinking Behavior/

71. exp Alcohol Deterrents/

72. exp Temperance/

73. ((alcohol or drunk$ or drink$) adj3 (consum$ or misus$ or abuse$ or intoxicate$ or harmful or excess$ or binge$ or hazardous$ or heavy or temperance or abstinence)).ab,ti.

74. (temperan$ or teetotal$).ab,ti.

75. or/70-74

76. (cognit$ adj2 stimulat$).ab,ti.

77. cognit$ exercis$.ab,ti.

78. (cognit$ adj2 (stimulat$ or train$ or exercis$)).ab,ti.

79. (brain adj2 (stimulat$ or train$ or exercis$)).ab,ti.

80. puzzle$.ab,ti.

81. crossword$.ab,ti.

82. reading.ab,ti.

83. (intellect$ adj2 activit$).ab,ti.

84. or/76-83

85. (socialis$ or socializ$).ab,ti.

86. (social$ adj2 (activit$ or stimulat$)).ab,ti.

87. (social adj3 (isolat$ or network$ or contact$ or alienat$)).ab,ti.

88. lonel$.ab,ti.

89. exp Loneliness/

90. or/85-89

91. 12 or 33 or 42 or 64 or 69 or 75 or 84 or 90

92. vision tests/

93. Eyeglasses/

94. vision disorders/

95. computer terminals/

96. Asthenopia/

97. ((sight or eyesight or vision or eye$) adj2 (protect$ or maintain$ or maintenance)).ti,ab.

98. or/92-97

99. hearing tests/

100. hearing loss/

101. hearing aids/

102. ((hearing or noise$) adj2 (protect$ or maintain$ or maintenance)).ti,ab.

103. or/99-102

104. 91 or 98 or 103

105. "sunburn".ab,ti.

106. Sunburn/

107. Sunscreening agents/

108. (sun adj2 (light or exposure or overexposure or screen$ or protect$ or tan$)).ab,ti.

109. vitamin D.ab,ti.

110. Vitamin D/

111. or/105-110

112. 104 or 111

113. (review or review,tutorial or review,academic).pt.

114. (medline or medlars or embase or pubmed or cochrane).sh,tw.

115. (scisearch or psychinfo or psycinfo).sh,tw.

116. (psychlit or psyclit).sh,tw.

117. cinahl.sh,tw.

118. (hand adj2 search$).sh,tw.

119. (manual adj2 search$).sh,tw.

120. "electronic database$".sh,tw.

121. "bibliographic databases".sh,tw.

122. "computeri?ed databases".sh,tw.

123. "online databases".sh,tw.

124. (pooling or pooled or "mantel haenszel").sh,tw.

125. or/114-124

126. 113 and 125

127. meta-analysis.sh,tw.

128. (meta-analys$ or meta analys$ or metaanalys$).sh,tw.

129. (systematic$ adj5 (review$ or overview$)).sh,tw.

130. (quantitativ$ adj5 (review$ or overview$ or synthes$)).sh,tw.

131. (methodologic$ adj5 (review$ or overview$)).sh,tw.

132. (integrative research review$ or research integration).tw.

133. or/127-132

134. 126 or 133

135. 112 and 134

136. Aged/

137. Retirement/

138. Aging/

139. Geriatrics/

140. older.ab,ti.

141. elder$.ab,ti.

142. senior$.ab,ti.

143. geriatr$.ab,ti.

144. retir$.ab,ti.

145. ag?ing.ab,ti.

146. longevity.ab,ti.

147. or/136-146

148. Middle Aged/

149. (middle adj ag$).ab,ti.

150. (baby adj2 boomer).ab,ti.

151. (midlife or "midlife" or mid-life or "mid life" or midlives or "mid lives" or mid-lives).ab,ti.

152. "middle life".ab,ti.

153. "later life".ab,ti.

154. ((ageing or aging) adj3 (well or success$ or positive$ or active$ or healthy)).ab,ti.

155. or/148-154

156. 147 or 155

157. 135 and 156

158. limit 157 to yr="2000 -Current"
