# Supplementary material for: Physical Activity in Community Dwelling Older People: A Systematic Review of Reviews of Interventions and Context
Source: PLoS One. 2016 Dec 20;11(12):e0168614. doi: 10.1371/journal.pone.0168614 (PMC5173028; doi:10.1371/journal.pone.0168614)
Supplement: S3 Protocol — (PDF) [file pone.0168614.s009.pdf]

PROSPERO International prospective register of systematic reviews

A systematic review of issues (barriers and facilitators) that prevent or limit or that help and motivate the uptake and maintenance of healthy behaviours in older people

Sarah Kelly, Louise Lafortune, Steven Martin, Nadja Smailagic, Andy Cowan, Carol Brayne

Citation

Sarah Kelly, Louise Lafortune, Steven Martin, Nadja Smailagic, Andy Cowan, Carol Brayne. A systematic review of issues (barriers and facilitators) that prevent or limit or that help and motivate the uptake and maintenance of healthy behaviours in older people. PROSPERO 2014:CRD42014015557 Available from
http://www.crd.york.ac.uk/PROSPERO_REBRANDING/display_record.asp?ID=CRD42014015557

Review question(s)

What issues (barriers and facilitators) prevent or limit or help and motivate the uptake and maintenance of healthy behaviours in older people (55+ years)?

Searches

A structured search strategy was developed using a wide range of search terms covering the following concepts and domains: ageing and older people; health behaviours and risk reduction relating to diet, physical activity, inactivity, alcohol, smoking; risk reduction relating to loneliness and isolation (i.e. leisure, social activities, participation), sun exposure, hearing and vision, dental health.

Databases searched include: MEDLINE, EMBASE, PsycINFO, CINAHL, Social Science Index, CENTRAL, The Cochrane Collaboration and Database of Systematic Reviews, Database of Abstracts of Reviews of Effects (DARE), HTA and York CRD databases. Relevant websites will be searched for grey literature (e.g. NHS Evidence, WHO, Open Grey, etc.). On going clinical trials registers to be searched to identify trials in progress. Finally, experts in the field will be consulted to identify any further potentially relevant papers.

As initial searches suggest a large volume of search hits, searching was conducted in two stages:

1) searching for systematic reviews in older age using a systematic review filter;

2) searching for published and on going primary studies in older age.

Systematic reviews and primary studies published from year 2000 onwards and published in English will be included. However, studies will not be excluded at the title/abstract screening stage on the basis of language so that the number of studies excluded on the basis of language can be measured and reported.

Only studies and systematic reviews that have aimed to include people in older age (55 years and over) living in the community will be included.

Types of study to be included

Only qualitative studies, systematic reviews or grey literature reports that include qualitative studies will be included in this review, using any type of research methods, including interviews, focus groups etc.

Where intervention studies or reviews of intervention studies also report qualitative data specific to barriers and facilitators to that intervention study or review, that data will be reported in the other two reviews of this series of reviews (see below).

Condition or domain being studied

Review of the issues (barriers and facilitators) that prevent or limit the uptake and maintenance of healthy behaviours in older people (55+ years). Modifiable behaviours include (but not exclusively): diet, physical activity, inactivity, alcohol, smoking, cognitive activity; risk reduction relating to loneliness and isolation (i.e. leisure, social activities;

participation), hearing and vision, dental health.

Participants/ population

Participants will be people aged 55 and over, living in the community, and would include:

• Healthy participants;

• People with pre-conditions for later ill health such as high blood pressure, high cholesterol, overweight or obesity, impaired cognitive function, mood disorders, functional limitations, impaired glucose tolerance (not limited to these conditions);

• People on medication as long as the medication did not limit their ability to fully take part in the health behaviour intervention of interest or directly affect the outcomes, or measurement of the data.

People from disadvantaged populations and minority groups, relating to health inequalities and vulnerable communities. Disadvantaged and minority populations will include (but is not limited to) low socioeconomic status, ethnic minority groups, LGBT groups, travellers and other groups with protected characteristics under the equality and diversity legislation.

Studies focused on people with previous ill health e.g. stroke, coronary heart disease, asthma (not limited to these conditions), will be excluded.

Intervention(s), exposure(s)

Studies looking at issues for people in older age that prevent or limit or which help and motivate them to:

• Increase/maintain levels of physical activity or decrease sedentary lifestyles or maintain balance, strength and weight-bearing functions;

• Improve/maintain good diet and nutrition (including components of diet e.g. fat intake, fruit and vegetable intake).

• Reduce/prevent/stop tobacco consumption;

• Decrease/prevent excessive alcohol consumption;

• Maintain/increase cognitive, leisure and social activities, and participation;

• Maintain hearing and vision;

• Improve/modify multiple behavioural risk factors;

• Prevent excessive sun exposure or increase sun exposure in those with inadequate exposure;

• Promote/improve dental health;

• Remove barriers/facilitate uptake and maintenance of any unhealthy/healthy behaviours with demonstration of impact;

Also included would be issues related to settings, mode of delivery and personnel:

• Community settings (including, but not limited to, home, workplace, community and day centres, sheltered housing, primary care);

• Interventions at individual, family, community, subnational or national level;

• Interventions in the private, public, voluntary or commercial sectors;

• Interventions delivered by healthcare professionals, lay people, home carers, researchers, media, Internet;

• Only interventions conducted in the countries of the Organisation for Economic Co-operation and Development (OECD) will be included.

Interventions in the following areas will be excluded:

• Use of prescription drugs/medication (except for medication available 'over the counter' such as nicotine patches or gum for smoking cessation);

• Use of dietary supplements;

• Management of existing disability, dementia, frailty and common non-communicable chronic disease;

• Management of obesity, including medical and surgical interventions for obesity;

• National policies, laws and taxation;

• Screening;

• Vaccination.

There will be no lower time limit for duration of intervention and follow-up.

Comparator(s)/ control

Any comparator or no comparator.

Context

Defined as above.

Outcome(s)

Primary outcomes

Primary qualitative outcomes from studies conducted in older age (55 and over years) relating to issues for people in older age (55 and over years) that prevent or limit or which help and motivate them to take up and maintain modifiable healthy behaviours that may impact on healthy ageing. Examples of healthy behaviours have been listed in the section above. Such issues may also include settings, mode of delivery and personnel issues.

Not applicable.

Secondary outcomes

None

Data extraction, (selection and coding)

Titles and/or abstracts will be screened independently by two reviewers using a decision form based on the inclusion criteria detailed in the review protocol. Differences between reviewers' results will be resolved by discussion and when necessary in consultation with a third reviewer. If after discussion, there is still doubt about the relevance of a study to the review it will be retained.

Full paper copies will be obtained for all reviews and studies identified by the title/abstract screening. A full paper screening tool with inclusion/exclusion criteria as defined in the review protocol will be developed for screening of the full papers. Full paper screening will be conducted independently by two people. Any differences of opinion about inclusion/exclusion will be resolved by discussion between the two reviewers or by consultation with a third reviewer.

Systematic reviews will be selected, quality assessed, and extracted first. Primary studies will then be selected, quality assessed, and extracted to supplement findings from SR (e.g. primary studies published after most recent high quality reviews), and to fill the gaps where no systematic reviews exist (e.g. specific behaviours, disadvantaged populations).

A flow chart will be used to summarise the number of papers included and excluded at each stage of the process. Systematic reviews and primary studies excluded at the full paper screening stage will be listed in the appendix of the review along with the reason for exclusion.

We will extract data on study design; participant characteristics and number; setting; type of outcome measures reported; outcome measures (barriers and facilitators to uptake and maintenance of healthy behaviours); A minimum of 10% of the studies will be fully double extracted (as below for quality assessment).

Risk of bias (quality) assessment

Study designs will be assigned using the methods used by NICE (glossary of study designs; appendix D, NICE methods manual: http://www.nice.org.uk/article/pmg4/chapter/appendix-d-glossary-of-study-designs) and the algorithm for classifying study designs (appendix E, NICE methods manual: http://www.nice.org.uk/article/pmg4/chapter/appendix-e-algorithm-for-classifying-quantitative-experimental-and-observational-study-designs).

Primary studies: When the type of intervention study design has been assigned, studies will be assessed for quality using the methods used by NICE (NICE methods manual appendices E through I: http://www.nice.org.uk/article/pmg4/chapter/1-introduction).

Systematic reviews: The methodological quality of each systematic review will be assessed using the AMSTAR tool (www.Amstar.ca). Each full review will be assessed by one reviewer and checked for accuracy by another. A minimum of 10% of the studies will be fully double assessed. Any discrepancy between reviewers would be resolved by discussion.

Each full paper will be assessed by one reviewer and checked for accuracy by another. A minimum of 10% of the studies will be fully double assessed. Any discrepancy between reviewers would be resolved by discussion.

Strategy for data synthesis

Findings will initially be tabulated to map the evidence in terms of study design, participants, intervention, setting and delivery, comparators, outcome measures (barriers and facilitators identified), firstly for systematic reviews to map the level of evidence, quality and gaps, and then for primary studies. Specific barriers and facilitators identified will be coded using qualitative analysis methods and analysed thematically (where sufficient data is available to identify themes). Otherwise, a descriptive approach to the available evidence will be taken.

Findings will be narratively synthesised and presented. Data specific to health inequalities and vulnerable communities will be assessed and findings may be summarised separately if sufficient data are available. Key themes based on analysis of the evidence across each health behaviour will be synthesised in a narrative format.

Evidence from systematic reviews and primary studies will be checked and any overlapping data will be reported to avoid over reporting of effect.

Analysis of subgroups or subsets

• Analysis will be conducted separately for each type of health behaviour reported.

• Disadvantaged and minority groups will be reported and analysed separately, if sufficient data are available

Dissemination plans

Findings from the review will be published in open access peer review journals. Outputs will be used to develop research activities within the Ageing Well Programme of the NIHR SPHR and CLAHRC for the East of England; and to inform public health guidance and practices.

Contact details for further information

Dr Kelly

Institute of Public Health

Forvie Site

School of Clinical Medicine

University of Cambridge

Box 113 Cambridge Biomedical Campus

Cambridge, CB2 0SR

sak65@medschl.cam.ac.uk

Organisational affiliation of the review
None

Review team
Dr Sarah Kelly, University of Cambridge
Dr Louise Lafortune, University of Cambridge
Mr Steven Martin, University of Cambridge
Dr Nadja Smailagic, University of Cambridge
Mr Andy Cowan, University of Cambridge
Professor Carol Brayne, University of Cambridge

Collaborators
Mr Gopal Kotecha, University of Cambridge, Medical School
Miss Vanda Ho, University of Cambridge, Medical School
Miss Krystine Kua, University of Cambridge, Medical School

Details of any existing review of the same topic by the same authors
Two complementary reviews are being carried in parallel by the review team. One focuses on the effectiveness of interventions in older age for increasing the uptake and maintenance of healthy behaviours that may impact on successful ageing (see PROSPERO protocol). Another review looks at the effectiveness of behavioural interventions in older age for the primary prevention, or delay, of dementia and cognitive decline (see PROSPERO protocol).

Anticipated or actual start date
10 November 2014

Anticipated completion date
31 December 2015

Funding sources/sponsors
This review is supported by the Ageing Well Programme of the National Institute for Health School for Public Health Research (NIHR SPHR) and the NIHR Collaborations for Leadership in Applied Health Research and Care (CLAHRC) for the East of England. No specific grant is linked to this project.

Conflicts of interest
None known

Other registration details
None

Language
English

Country
England

Subject index terms status
Subject indexing assigned by CRD

Subject index terms
Aged; Aged, 80 and over; Health Behavior; Health Promotion; Humans; Lifestyle

Stage of review
Ongoing

Date of registration in PROSPERO
17 December 2014

Date of publication of this revision
17 December 2014

DOI
10.15124/CRD42014015557

Stage of review at time of this submission	Started	Completed
Preliminary searches	Yes	No
Piloting of the study selection process	Yes	No
Formal screening of search results against eligibility criteria	Yes	No
Data extraction	No	No
Risk of bias (quality) assessment	No	No
Data analysis	No	No

PROSPERO
International prospective register of systematic reviews
The information in this record has been provided by the named contact for this review. CRD has accepted this information in good faith and registered the review in PROSPERO. CRD bears no responsibility or liability for the content of this registration record, any associated files or external websites.
